# The Roles of Composition and Mesostructure of Cobalt‐Based Spinel Catalysts in Oxygen Evolution Reactions

**DOI:** 10.1002/chem.202102400

**Published:** 2021-10-22

**Authors:** Anna Rabe, Julia Büker, Soma Salamon, Adarsh Koul, Ulrich Hagemann, Joachim Landers, Klaus Friedel Ortega, Baoxiang Peng, Martin Muhler, Heiko Wende, Wolfgang Schuhmann, Malte Behrens

**Affiliations:** ^1^ Faculty of Chemistry University of Duisburg-Essen and Center for Nanointegration Duisburg-Essen (CENIDE) Universitätsstr. 7 45141 Essen Germany; ^2^ Laboratory of Industrial Chemistry Faculty of Chemistry and Biochemistry Ruhr University Bochum Universitätsstr. 150 44780 Bochum Germany; ^3^ Faculty of Physics and CENIDE University of Duisburg-Essen Lotharstraße 1 45057 Duisburg Germany; ^4^ Analytical Chemistry-Center for Electrochemical Sciences (CES) Faculty of Chemistry and Biochemistry Ruhr University Bochum Universitätsstr. 150 44780 Bochum Germany; ^5^ Interdisciplinary Center for Analytics on the Nanoscale (ICAN) University of Duisburg-Essen Carl-Benz-Straße 199 47057 Duisburg Germany; ^6^ Center for Nanointegration Duisburg-Essen (CENIDE) Carl-Benz-Straße 199 47057 Duisburg Germany; ^7^ Institute for Inorganic Chemistry Christian-Albrechts-Universität zu Kiel Max-Eyth-Str. 2 24118 Kiel Germany

**Keywords:** cobalt spinels, co-precipitation, crystalline precursor decomposition approach, oxygen evolution reaction, structure-reactivity relationship

## Abstract

By using the crystalline precursor decomposition approach and direct co‐precipitation the composition and mesostructure of cobalt‐based spinels can be controlled. A systematic substitution of cobalt with redox‐active iron and redox‐inactive magnesium and aluminum in a cobalt spinel with anisotropic particle morphology with a preferred 111 surface termination is presented, resulting in a substitution series including Co_3_O_4_, MgCo_2_O_4_, Co_2_FeO_4_, Co_2_AlO_4_ and CoFe_2_O_4_. The role of redox pairs in the spinels is investigated in chemical water oxidation by using ceric ammonium nitrate (CAN test), electrochemical oxygen evolution reaction (OER) and H_2_O_2_ decomposition. Studying the effect of dominant surface termination, isotropic Co_3_O_4_ and CoFe_2_O_4_ catalysts with more or less spherical particles are compared to their anisotropic analogues. For CAN‐test and OER, Co^3+^ plays the major role for high activity. In H_2_O_2_ decomposition, Co^2+^ reveals itself to be of major importance. Redox active cations in the structure enhance the catalytic activity in all reactions. A benefit of a predominant 111 surface termination depends on the cobalt oxidation state in the as‐prepared catalysts and the investigated reaction.

## Graphical abstract

**The particle anisotropy** and cobalt content in spinel oxides is systematically varied and the impact on oxygen evolving reactions is investigated. For chemical and electrochemical water oxidation as well as H_2_O_2_ decomposition the reactivity changes with the cobalt‐to‐metal ratio and the degree of anisotropy. Furthermore, the importance of the interplay of redox pairs and different active sites are highlighted.

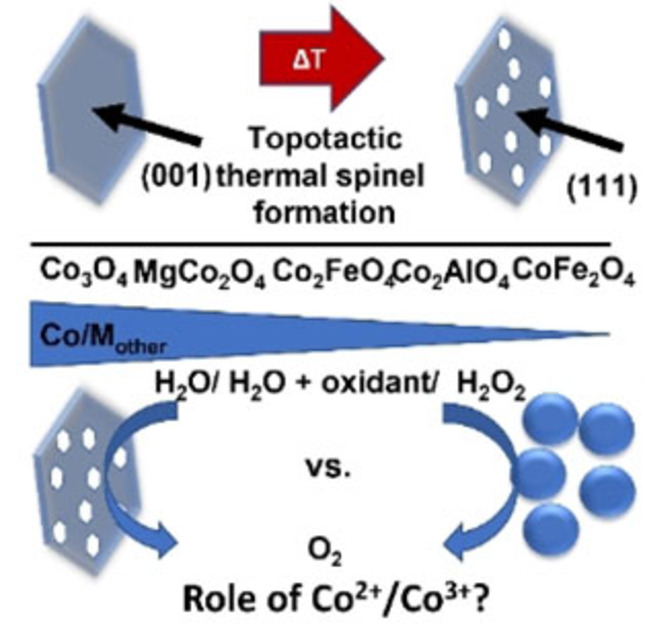

## Introduction

Heterogeneous oxidation catalysis over mixed metal oxides depends critically on their composition and also properties like particle size, particle morphology, and porosity‐here summarized as mesostructure‐play an important role.^[1]^ Applied catalysts usually have complex multi‐cation compositions that are specifically optimized for a given process.^[2]^ However, systematic studies including different reactions over the same catalyst and comprehensive knowledge of real structure‐reactivity correlation are scarce and have the potential to reveal general trends in heterogeneous catalysis of mixed oxides. Therefore, the herein presented work systematically investigates the impact of composition and mesostructure of cobalt‐based spinel oxide catalysts on a variety of oxidation reactions.

Spinel oxides are an attractive playground for such studies as isomorphous substitution of transition as well as main group metal cations is possible while allowing single‐phase materials. The normal spinel oxide structure has the generic formula (A^II^_*t*_)(B^III^_*o*_)_2_O_4_ consisting of cubic closed packed oxygen atoms with the B metal cations occupying half of the octahedral sites (index *o*) and the A atoms occupying one eighth of the tetrahedral sites (index *t*).^[3]^ Already countless investigations of their general properties have been conducted and applications as for example Li‐ion battery materials,^[4]^ super capacitors,^[5]^ and electro catalysts^[6]^ have been reported. Sojka et al. have recently reported an example for the potential of a comparative study of a spinel isomorphous substitution series for determining the roles of different cations in catalytic N_2_O decomposition. They were able to identify Co^3+^ in octahedral sites as active site for this specific reaction.^[7]^ The same group investigated the activity of different supported cobalt‐based spinel catalysts in the electrochemical oxygen reduction reaction (ORR). For example, they found the ratio of exposed facets to be of importance for the activity of a manganese‐cobalt spinel on various carbon supports.^[8]^ In addition they showed the influence of composition and especially phase purity for the selectivity and activity of mixed iron cobalt spinels.^[9]^ Using systematic cation substitution and tuning of the catalysts mesostructure with constant cation ratios, we herein exploit and extend this powerful approach to different oxygen evolving reactions.

Spinel oxides can be synthesized by various approaches, for example direct co‐precipitation,^[10]^ sol‐gel method,^[11]^ hydro‐ and solvothermal synthesis,^[12]^ nanocasting,^[1c]^ or solid state reactions.^[13]^. Most of these synthesis methods result in mostly isotropic particles or contain organic residues, which complicate structure‐reactivity investigations. We recently reported the synthesis of an anisotropic cobalt ferrite spinel using the crystalline precursor decomposition approach.^[14]^ With this technique anisotropic phase pure mixed spinels without organic surfactants can be reproducibly synthesized. As crystalline precursors hydroxides as well as layered double hydroxides (LDH), both typically crystallizing in a platelet‐like morphology, turned out to be the most convenient for several reasons.

Layered double hydroxides are so called hydrotalcite‐like materials with the general formula [M_1‐x_^2+^M_x_^3+^(OH)_2_]^x+^[A_x/n_]^n−^ ⋅ xH_2_O. These compounds consist of brucite‐like M(OH)_2_ layers, with a fraction of bivalent cations substituted by trivalent cations. The resulting excessive charge is compensated by intercalation of anions between the layers leading to strong electrostatic interactions connecting the positively charged brucite layers with the anionic inter‐layers. In addition, water molecules are intercalated in the layered structure and form hydrogen bonds.^[15]^ These materials usually exhibit a well‐defined hexagonal platelet morphology. A vast variety of cations M and anions A, in air usually carbonate, can be built into the structure and a homogeneous cation distribution is found.^[16]^ The crystalline nature assures a high reproducibility of the as‐prepared hydroxide precursors. A limiting factor for phase pure LDH formation is x, representing the ratio of bivalent to trivalent cations. x should not exceed 0.4, otherwise by‐phase formation is observed.^[17]^

If a fraction of the incorporated cations can be oxidized upon thermal treatment the needed cation stoichiometry M^2+^/M^3+^ of 1 : 2 for spinel formation can be achieved through partial oxidation of divalent to trivalent cations when decomposing the LDH precursor. Through mild calcination temperatures a topotactic transformation along the (001)_LDH_I(111)_Spinel_ planes without significant diffusion of the cations occurs.^[18]^ The resulting spinels are pseudo‐morphs of the precursor hydroxides, retaining the unusual and unique hexagonal platelet morphology and defined cation distribution. In addition, the thermal treatment leads to decomposition of the intercalated carbonate and water creating pores within the platelet structure, maximizing the surface area of the formed calcination products. Because of the topotactic transformation a dominant 111 surface termination is formed, making spinels synthesized by this approach perfect candidates for investigating structure‐reactivity correlations.[^14^, ^18^]

With a systematic substitution series of bivalent and trivalent cations in the precursor structure with redox active and redox inactive metals the composition and the ratio of Co^2+^/Co^3+^ is systematically varied. As a starting point the pure cobalt hydroxide is used and substituted with the redox active transition metal iron and redox inactive, meaning a fixed oxidation state, bivalent and trivalent main group elements magnesium and aluminum. The resulting anisotropic cobalt spinel substitution series is Co_3_O_4_, MgCo_2_O_4_, Co_2_FeO_4_, Co_2_AlO_4_ and CoFe_2_O_4_.

In addition to investigating the impact of composition, especially the interplay of redox pairs, the effect of morphology and its anisotropy is examined. Therefore, isotropic Co_3_O_4_ and CoFe_2_O_4_ is synthesized via a crystalline precursor and direct co‐precipitation, respectively, for comparison. These isotropic particles are assumed to not have a predominant surface termination and therefore show differences in catalytic activity compared to their analogs with a predominant 111 surface termination.

Herein we extend our previous work on the crystalline precursor decomposition approach to new compositions and combine it with the above‐mentioned isomorphous substitution study to learn about the role of the different cations and morphology in different oxygen evolving reactions. The investigated reactions are chemical water oxidation, using Ce^4+^ as an oxidizing agent (CAN: cerium ammonium nitrate). This reaction is mainly governed by the exposed surface of the catalysts and is established as a probe reaction for water oxidation on other catalysts.^[19]^ Electrochemical oxygen evolution reaction (OER) in alkaline medium is performed, requiring additional charge transport through the catalyst layers by electric conductivity of the samples. Due to its sluggish kinetics, it is considered the bottleneck for electrochemical hydrogen and oxygen production and therefore a crucial reaction to be investigated in the energy context.^[20]^ As a comparatively facile oxygen evolving reaction, H_2_O_2_ decomposition is conducted as a third probe reaction. Decomposition of peroxide species represents a potential intermediate step for oxygen evolution from water and is of relevance for Fenton‐like oxidation reactions.

## Results and Discussion

A systematic substitution series of cobalt containing spinel catalysts was synthesized using the crystalline precursor decomposition approach. The precipitation reactions were performed under highly controlled conditions, leading to repetitious accuracy of the synthesized catalysts’ properties. Further information on reproducibility can be found in the Supporting Information (Figure S1–S3). The as‐prepared precursors were labeled according to their relative nominal metal cation composition and hydroxide type, the spinel catalysts were labeled by their nominal metal cation composition. To differentiate the isotropic Co_3_O_4_ and CoFe_2_O_4_ with spherical particles from their anisotropic analogs with platelet morphology, the prefix iso‐ is introduced.

## Precursor characterization

**Figure 1:**
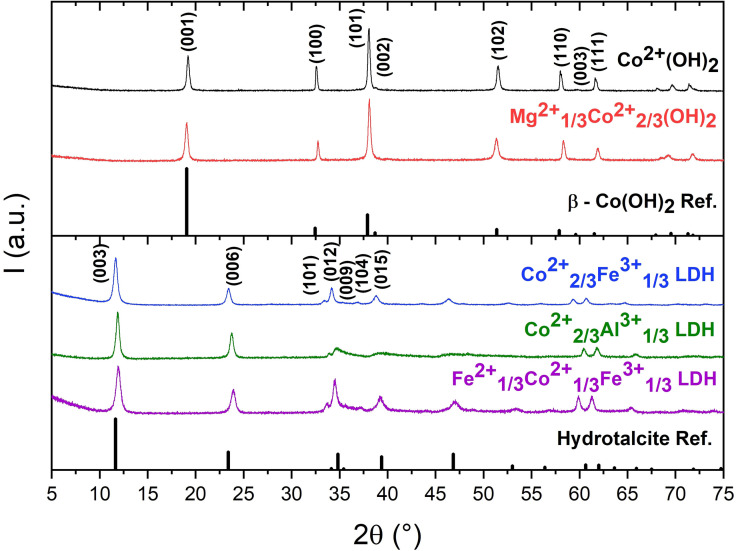
PXRD patterns of the as‐prepared hydroxide (top), layered double hydroxide (bottom) precursors and the corresponding reference patterns of β‐Co(OH)_2_ (ICSD No. 88940) and Hydrotalcite (ICSD. No. 6296).

The five hydroxides and layered double hydroxides acting as crystalline precursors for the anisotropic substitution series were co‐precipitated and thoroughly characterized. Synthesis protocols demonstrating the controlled synthesis conditions can be found in Figure S4 and full details on precipitation conditions are given in the experimental section. Figure 1 shows the PXRD patterns proving phase purity of the as‐prepared precursors. For the Fe^2+^_1/3_Co^2+^_1/3_Fe^3+^_1/3_ LDH Mössbauer spectroscopy and X‐ray photoelectron spectroscopy (XPS) were performed to analyze the metal cation oxidation states in detail. The results and discussion can be found in the Supporting Information (Figure S5). All precipitated materials exhibit a mostly hexagonal platelet morphology with varying aspect ratios depending on the composition (Figure S6). The cationic sub‐lattices of these as‐prepared crystalline precursors function as templates for the desired pseudo‐morphic spinel catalysts.

**Figure 2:**
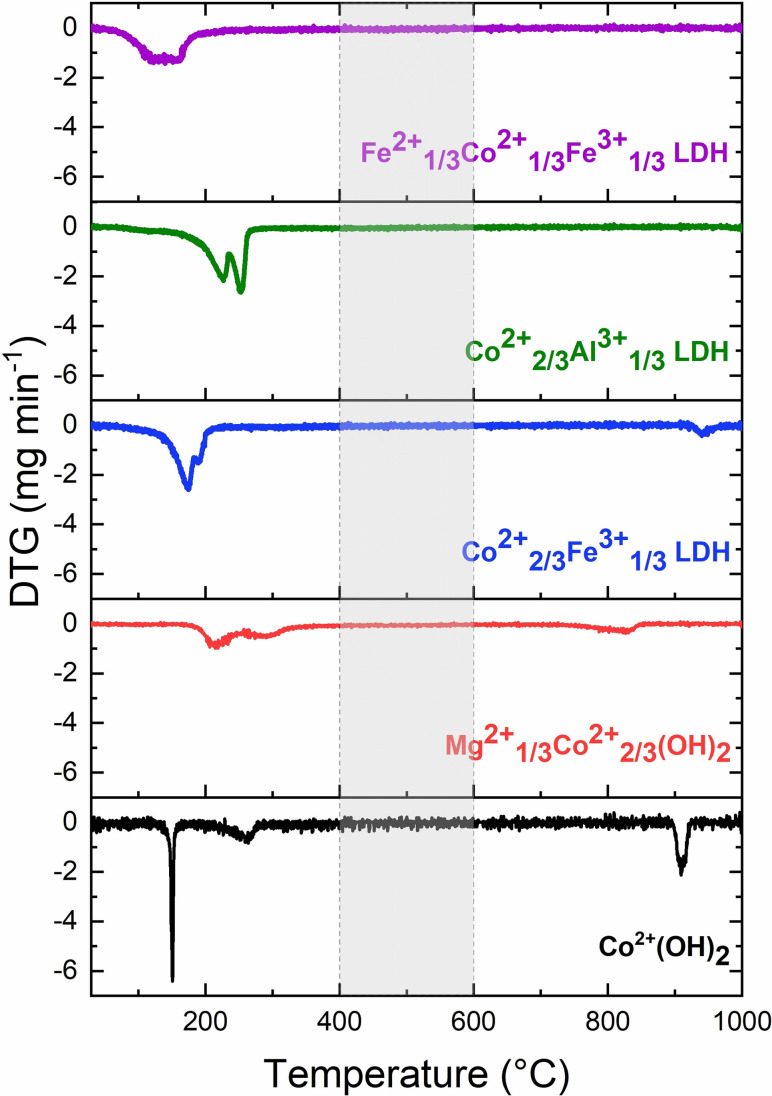
DTG curves resulting from the thermogravimetric analysis of the precursors. In the temperature range highlighted in grey no mass loss occurs and calcination temperatures were therefore chosen within this range.

To investigate the behavior of the precursors upon thermal treatment, thermogravimetric analysis was performed. All precursors show the expected behavior upon thermal treatment as reported in literature. Figure 2 shows the derivative thermogravimetric (DTG) curves, ensuring complete precursor decomposition at 400 °C, which was chosen as calcination temperature for most samples. The relative mass loss curves are shown in Figure S7. For cobalt hydroxide and cobalt magnesium hydroxide the dominant weight loss step is assigned to dehydroxylation. The layered double hydroxides show a typical twostep decomposition consisting of dehydroxylation and decarboxylation.^[21]^ For the cobalt spinel, magnesium cobaltite and iron cobaltite, an additional mass loss at 909 °C, 826 °C and 942 °C respectively, is observed. This can be explained by the thermal reduction of Co(III) oxide to Co(II) oxide confirming the presence of trivalent Co(III) in these materials.^[22]^ Co_2_AlO_4_ and CoFe_2_O_4_ do not show this additional thermal reduction step as the spinel structure with these compositions is stable up to higher temperatures investigated here.^[23]^

Thermogravimetric analysis suggests a sufficient calcination temperature of 400 °C for all samples except CoFe_2_O_4._ For this composition, a small amount of a hematite by‐phase was observed upon thermal treatment at 400 °C but vanished at 600 °C. A more detailed discussion and characterization of the respective samples can be found in the Supporting Information (Figure S8). To not only investigate the impact of composition but that of morphology isotropic Co_3_O_4_ and CoFe_2_O_4_ were synthesized. In case of Co_3_O_4_ a cobalt hydroxy carbonate was precipitated and thermally oxidized to a cobalt spinel. Isotropic CoFe_2_O_4_ was directly precipitated and thermally treated to trigger crystallization of potential X‐ray amorphous by‐phases. Further experimental details are available in the experimental section. Synthesis protocols, PXRD patterns, SEM micrographs and TGA results can be found in the Supporting Information (Figure S9–S12).

The as‐prepared anisotropic and isotropic precursors were thoroughly characterized and subsequently oxidized to the desired spinel catalysts.

## Catalyst characterization

**Figure 3:**
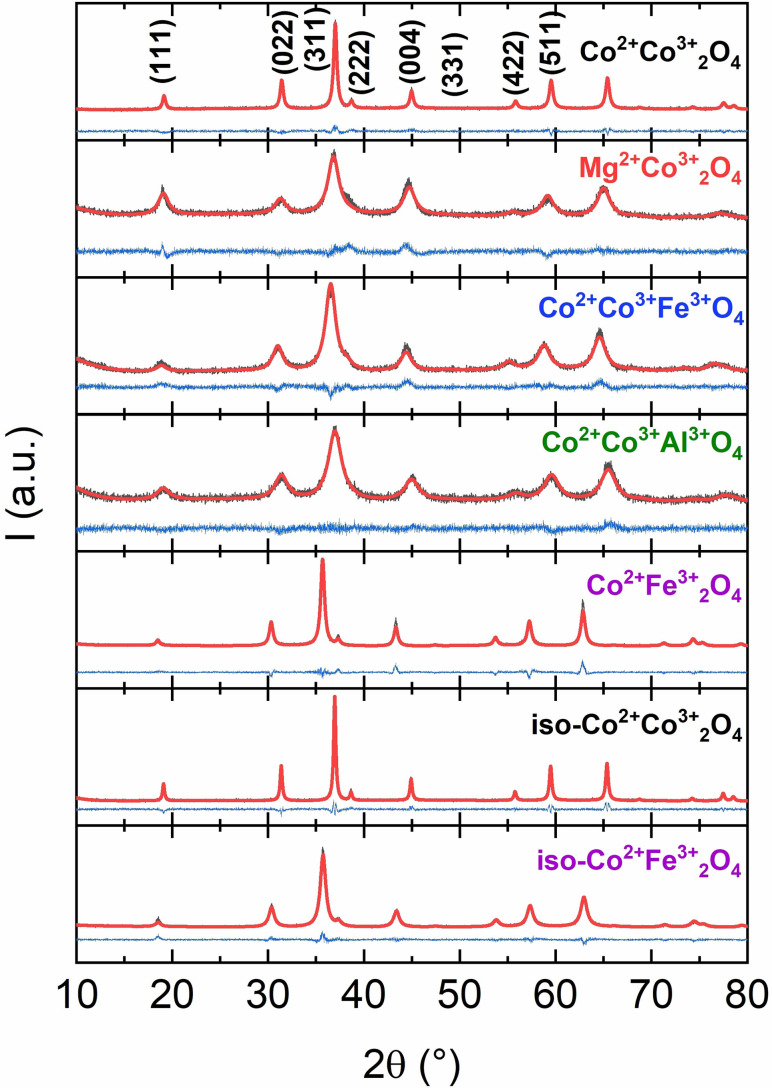
Rietveld refinements of the anisotropic cobalt spinel substitution series and isotropic Co_3_O_4_ and CoFe_2_O_4_. Measured data is shown in grey, the calculated pattern in red and the difference plot in blue. The main reflections of the cubic spinel phase were labelled.

Upon calcination the targeted substituted cobalt spinel catalysts were formed. Figure 3 displays the PXRD patterns of the substitution series, confirming at first sight only single spinel phases present. In case of Co_2_FeO_4_ the phase diagram predicts a miscibility gap with phase segregation into a cobalt richer and an iron richer spinel phase.^[23]^ The establishment of phase purity is not straightforward based alone on PXRD data of this nanostructured sample. This aspect is in the focus of our forthcoming work, and we assume segregation into two phases according to the phase diagram. Here in this contribution, we use a two‐spinel phase model for the refinement of the X‐ray data of this sample. Rietveld refinement further underlines the phase purity of the other samples and the absence of non‐spinel phases in all samples. The determined lattice parameters (Table 1) agree with literature values. The domain sizes were extracted from Rietveld refinement as well. Due to the quite low calcination temperatures the absolute values of the domain sizes are low. The peak width of the diffractograms fit the determined values perfectly. The most defined diffraction patterns show the highest crystallinity and vice versa. Rietveld fit parameters are listed in Table S1.

**Table 1:** Lattice parameters, domain sizes, surface areas, nominal and experimental cobalt to metal ratios of the anisotropic cobalt spinel substitution series and the isotropic Co_3_O_4_ and CoFe_2_O_4_.

Sample	Lit. lattice parameter [Å]	Lattice parameter [Å] ^[a]^	Domain size [nm]^[b]^	Surface area [m^2^ g^−1^]^[c]^	Nominal Co/M	Exp. Co/M^[d]^
Co_3_O_4_	8.065^[24]^	8.078	15.0	30	–	–
MgCo_2_O_4_	8.107^[25]^	8.130	4.3	50	2	2.33±0.43
Co_2_FeO_4_	8.242^[26]^	8.130 8.171	5.8 4.6	90	2	1.80±0.04
Co_2_AlO_4_	8.087^[27]^	8.058	3.5	176	2	2.38±0.44
CoFe_2_O_4_	8.394^[26]^	8.376	12.8	28	0.5	0.46±0.01
iso‐Co_3_O_4_	8.065	8.079	23.2	30	–	–
iso‐CoFe_2_O_4_	8.394	8.366	8.8	40	0.5	0.50±0.01

[a] Derived from Rietveld refinement. All samples consist of a cubic spinel phase with the space group Fd3 m. a=b=c. [b] The domain size was determined as the volume‐weight mean column height from integral breadth. [c] Calculated with the BET method. [d] Based on XRF of the spinel catalysts. The error was determined by error propagation using the uncertainty specified by the instrument.

The textural properties of the spinels were investigated with nitrogen physisorption experiments and BET theory. Surface areas of the catalysts are listed in Table 1 and vary significantly with composition. Nevertheless, all samples show the expected mesoporosity. Complete adsorption desorption isotherms and pore size distributions of the spinels and their precursors are shown in Figure S13 and 14.

**Figure 4:**
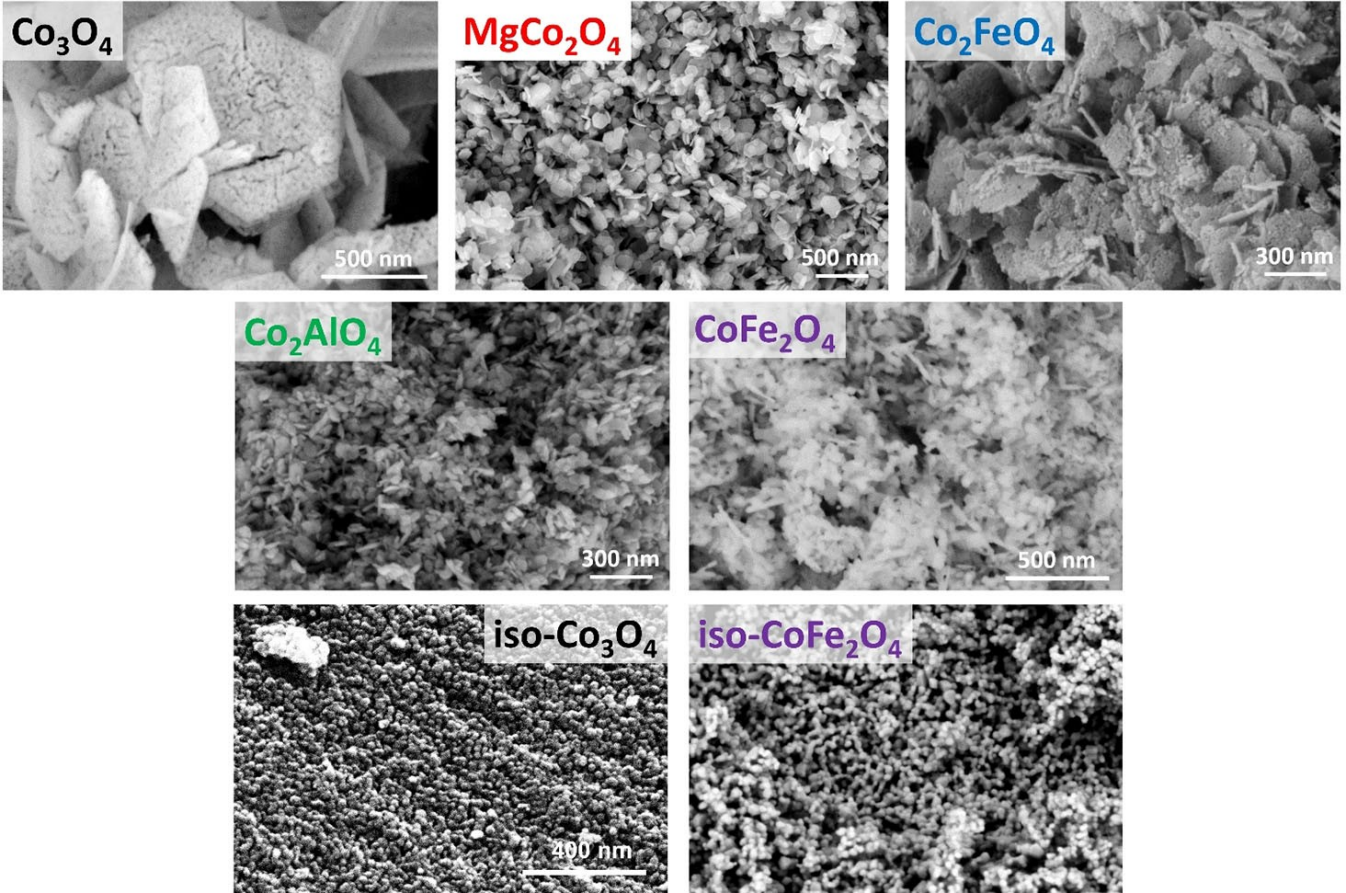
SEM images of the as‐prepared spinel substitution series.

**Figure 5:**
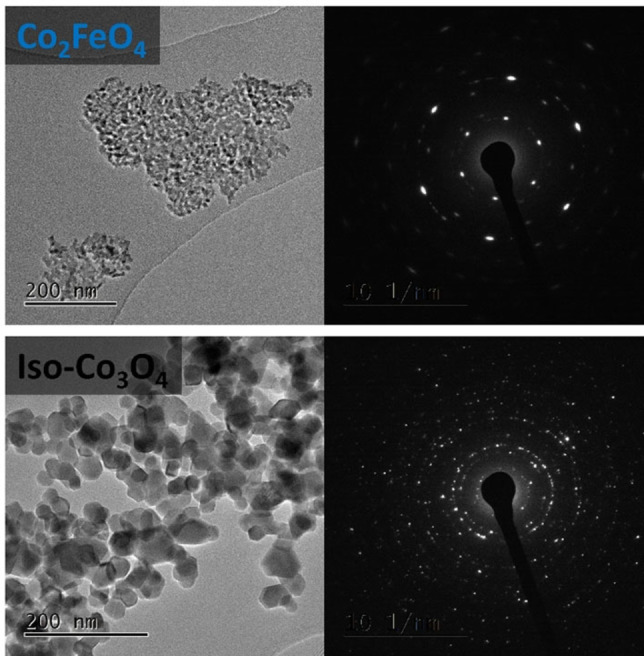
SAED and TEM micrographs of iso‐Co_3_O_4_ and Co_2_FeO_4_ showing the ordered orientation of the spinel crystallites within one platelet in the former and the disordered orientation in the latter sample. The zone axis is [111] relative to the spinel structure suggesting together with the platelet orientation perpendicular to the viewing direction a predominant 111 termination of the lateral surfaces of the platelets.

The SEM micrographs in Figure 4 give an overview of the preserved anisotropic morphology for the substitution series dictated by the (layered double) hydroxide precursors, which is contrasted by the isotropic Co_3_O_4_ and CoFe_2_O_4_ particles. Even though the degree of anisotropy, in other words aspect ratio, differs within the anisotropic samples, all catalysts of this substitution series clearly exhibit a hexagonal platelet‐like morphology. Due to the decomposition of the anions in the layered precursor structure, holey platelets are formed, increasing the surface area of the catalysts. A comparison of the precursor surface areas and the spinels is shown in Figure S15. Only the anisotropic CoFe_2_O_4_ sample shows a decrease in surface area compared to its precursor. This is explained by the higher calcination temperature required to remove the hematite by‐phase leading to sintering of the pores. In Figure S16 TEM images of the particles are shown confirming the porous and platelet morphology of the substitution series. As mentioned in the introduction, the transformation from the anisotropic precursor to the cubic spinel was previously found to occur topotactically, leading to a predominant 111 termination on the lateral surface of the platelets. This was shown for CoFe_2_O_4_ and Co_3_O_4_ synthesized by a similar technique by us and others.[^14^, ^18^] In Figure 5 the electron diffraction pattern of a single Co_2_FeO_4_ flake from this work is shown, underlining this statement also for this composition.

The diffraction pattern resembles one that would be expected for a single crystal with a viewing direction along [111]. Together with the knowledge of the platelet‐like particle morphology and its orientation perpendicular to the electron beam, a predominant 111 termination can be concluded. The TEM images of the isotropic spinels give rise of a few small platelet‐like structures for the Co_3_O_4_ sample, most likely due to Co(OH)_2_ by‐phase formation during the precipitation of the precursor. Nevertheless, the isotropy of this sample is far more pronounced than for its anisotropic counterpart derived from a hydroxide structure. The selected area electron diffraction pattern clearly shows the transition towards a ring pattern indicating a more statistical distribution of the crystallite's orientations. Based on their more spherical shape, the isotropic spinels are thus expected to exhibit a less predominant surface termination, which is less affected by their preparation history compared to the anisotropic materials derived from the crystalline precursor decomposition approach. Furthermore, first principles calculations and inverse Wulff constructions of Co_3_O_4_ nanoparticles emphasize a surface termination with several low indexed facets like 100, 110, 112 and 111.^[28]^ The TEM images of all samples are shown in the Supporting Information. Due to the intergrown nature of the materials, it was not possible to obtain electron diffraction on individual platelets in all cases. As a consequence, spotty diffraction patterns with different tendencies towards diffraction rings were obtained and the evidence for the preferred 111 termination of the platelets was not as clearly established as for Co_2_FeO_4_ (Figure 5) or in previous literature reports for Co_3_O_4_ and CoFe_2_O_4_.[^14^, ^18^] However, based on the indications discussed in the Supporting Information, we assume the preferred 111 termination for all our samples relative to the iso‐catalysts and will discuss the catalytic properties in the light of this assumption. Particle size distributions of all catalysts were estimated based on the electron microscopy data which is shown in Figure S17 and were found to be monomodal, but scatter between 61±13 nm (Co_2_AlO_4_) and 1147±309 nm (Co_3_O_4_) for the anisotropic and between 18±6 nm (CoFe_2_O_4_) and 29±8 nm (Co_3_O_4_) for the isotropic spinel particles.

In conclusion, the anisotropic cobalt spinel substitution series as well as the isotropic cobalt spinel and cobalt ferrite were thoroughly characterized. They consist only of spinel phases and exhibit the expected morphology differences between anisotropic and isotropic samples. The specific surface areas were analyzed and found to cover a wide range from 28 to 176 m^2^/g. These values will be used to normalize the kinetic results to allow an accurate comparison of the catalytic testing, which is presented in the following paragraphs.

## Catalysis

The prepared substituted cobalt spinel series as well as the isotropic cobalt spinel and cobalt ferrite were tested for their performance in chemical water oxidation using Ce^4+^ as an oxidizing agent (CAN test), electrochemical oxygen evolution reaction (OER) under alkaline conditions and H_2_O_2_ decomposition. Here, we discuss the activity trends in the three probe reactions in the context of the spinel composition, with a focus on the total cobalt content and the relative amount of Co^3+^, and the comparison between the anisotropic and isotropic Co_3_O_4_ and CoFe_2_O_4_ samples.

**Figure 6:**
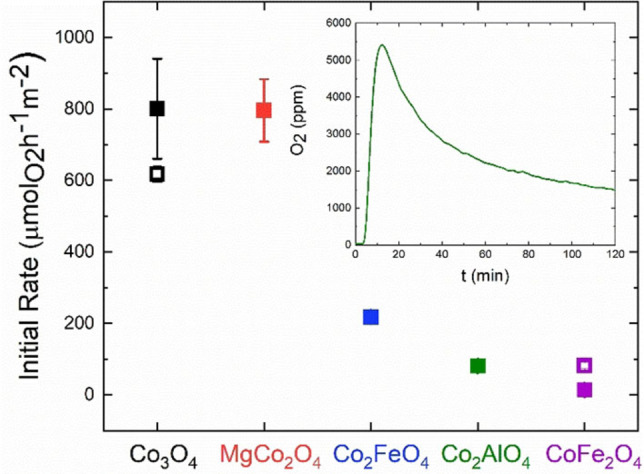
Initial rates for the CAN test of the anisotropic cobalt spinel substitution series (closed symbols) and the isotropic samples (open symbols). Error bars result from twofold measurements. For anisotropic Co_2_FeO_2,_ Co_2_AlO_4_, CoFe_2_O_4_ and the isotropic spinels the error bars do not exceed the symbol sizes. The inset shows the oxygen evolution per time unit over 2 h for Co_2_AlO_4_ as an example.

Figure 6 shows the initial rates of the substitution series and the isotropic samples for the CAN test after normalization by BET surface area, and the total oxygen evolution over 2 h for Co_2_AlO_4_ as an example in the insert. The total oxygen evolution for the other samples is shown in Figure S18. The CAN test has been used as a probe reaction for OER with the important difference that the purely chemical driving force by the oxidation potential of the dissolved Ce^4+^ is exposed uniformly to the immersed catalyst's surface and the activity is thus less affected by transport phenomena, binder additives, electrical contact problems or electrochemical ensemble effects compared to electrochemical testing.^[19a]^

Co_3_O_4_ and MgCo_2_O_4_ both have a nominal 2 : 1 ratio of Co^3+^: M^2+^ (M=Co, Mg) and exhibit the same and the highest activity suggesting that the role of Co^2+^ is only minor and can be played as well by the redox‐inert Mg^2+^. Upon substitution of half of the Co^3+^ cations in Co_3_O_4_ with Fe^3+^ or Al^3+^, a significant decrease in activity is observed, highlighting again the critical catalytic role of this cation. In addition, Co_2_AlO_4_ containing the non‐redox active cation Al^3+^ shows a smaller initial rate than its iron containing analog Co_2_FeO_4_. This observation is in line with the reports that iron, in contrast to aluminum, can act as a promoter for oxygen evolution in cobalt or nickel‐based materials.^[29]^ This finding supports the importance of the interplay in the electronic structure of oxidation catalysts, in short, the redox pairs involved in the catalytic cycle.^[30]^ However, also a phase separation into an iron rich and cobalt rich spinel as discussed earlier (see Table 1), has to be taken into account, which could give rise to an activity intermediate between Co_3_O_4_ and CoFe_2_O_4_. Cobalt ferrite shows the lowest activity in the series, underlining again the importance of Co^3+^ for the CAN‐induced chemical oxygen evolution reaction.

As exposed surface facets and catalytic performance are related, a difference in activity is anticipated and observed for the samples with same composition and different morphology. The predominantly 111 terminated Co_3_O_4_ performs better than its isotropic analog, suggesting that Co^3+^ might be especially effective if residing in this facet. However, the isotropic CoFe_2_O_4_ shows an enhanced catalytic activity compared to the 111 terminated cobalt ferrite, it even shows a comparable initial rate to the Co^3+^ containing Co_2_AlO_4._ Hence preferred orientation does not seem to play a significant positive role if no trivalent cobalt is present.

**Figure 7:**
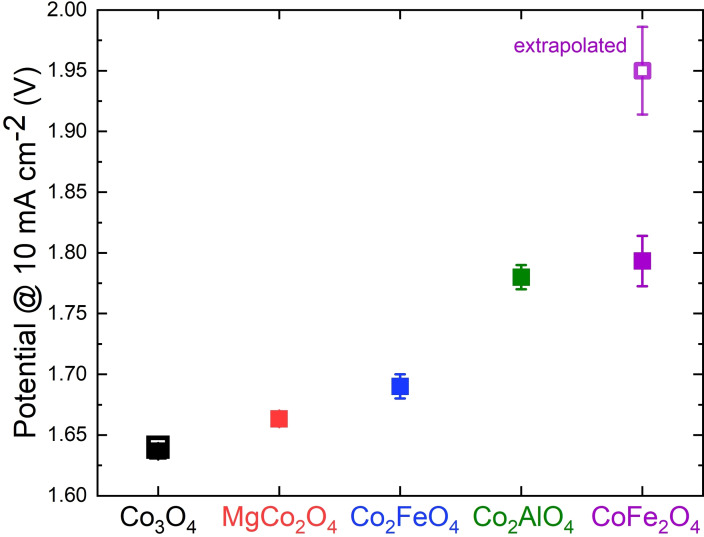
Measured potential at 10 mA cm^−2^ normalized by the geometric area of the electrode for the anisotropic substitution series (closed symbol) and the isotropic samples (open symbols). 10 mA cm^−2^ was chosen as point for comparison based on the suggestion by McCrory et al.^[33]^ The data point for isotropic cobalt ferrite was extrapolated, as it did not reach 10 mA cm^−2^ in the applied potential range. Error bars result from three independent measurements.

Electrochemical oxygen evolution was conducted for the whole anisotropic series and the isotropic analogs after drop casting the powder on glassy carbon electrodes. Recent investigations showed the transformation of cobalt‐based catalysts during OER. Independent of the initial oxidation state of cobalt, a CoO_x_(OH)_y_ is reversibly formed as active phase at applied anodic potentials required for the OER.^[31]^ Nevertheless, the activity correlates with other cations incorporated in the structure due to different stabilization abilities of surface species of the formed oxyhydroxides.^[32]^ The current density as a function of the applied potential normalized by the geometric area of the electrode are shown in Figure S19 and the corresponding potentials at 10 mA cm^−2^ are shown in Figure 7. They exhibit the same distinct dependence on the composition as found for the CAN test.

Furthermore, the isotropic cobalt oxide shows a slightly higher overpotential for the OER than its anisotropic equivalent. On the contrary, the isotropic cobalt ferrite showed an extreme increase in overpotential, not even reaching 10 mA cm^−2^ at the highest applied potential. In case of Co_3_O_4_ the preferred orientation does not play a significant role, while for the Co^3+^‐free cobalt ferrite it indeed does.

To address the topic of normalization in electrochemical investigations with rotating disk electrodes the current densities were further normalized by the BET surface area of the catalysts (Figure S19). In general, the trend is preserved with one exception. The high surface area of the Co_2_AlO_4_ leads to a decrease in the performance upon BET area normalization, causing an apparent higher catalytic activity of the anisotropic cobalt ferrite. The double layer capacitances C_dl_ of the catalysts were determined as well as it scales with the electrochemically active surface area (ECSA).^[34]^ Details of the procedure for C_dl_ determination are given in the experimental section. Figure S21 shows an exemplary procedure of the determination for the anisotropic Co_3_O_4_ catalyst. The potential at 10 mA cm^−2^ as a function of C_dl_ is shown in Figure S20. As different compositions in spinels have an enormous impact on C_DL_, a normalization by double layer capacitance is not feasible for the substitution series with different elements.^[35]^ However, the comparison of samples with the same composition is reasonable and gives further insight in possible structure‐reactivity relationships. The two cobalt spinels exhibit nearly the same overpotential, but a significantly different value for C_dl_. The two cobalt ferrites on the other hand show nearly the same C_dl_ but very different activities. This underlines the importance of the mesostructure for this reaction, as differences or similarities in activity cannot simply be explained by variation of the ECSA.

**Figure 8:**
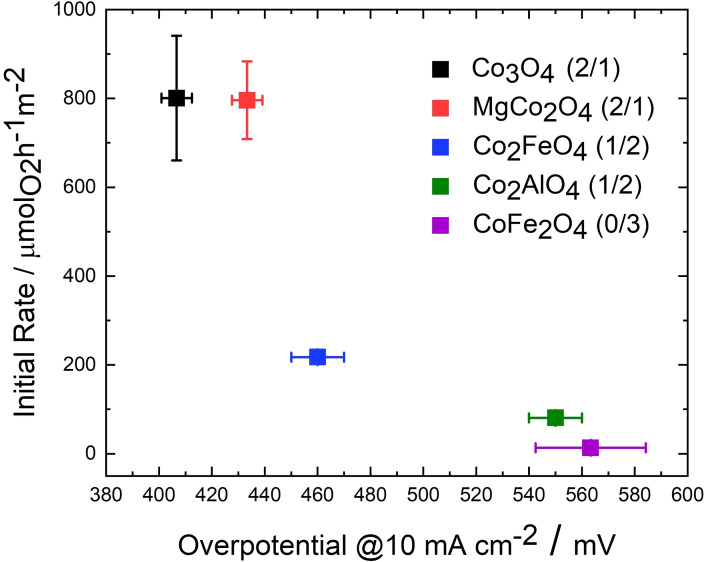
Initial rates for chemical water oxidation (CAN test) vs. the overpotential for electrochemical OER at 10 mA cm^−2^ for the cobalt spinel substitution series. The ratio Co^3^/M_others_ is specified in brackets. Error bars for the overpotential are determined from three independent measurements.

Figure 8 shows the initial rates in the CAN test plotted against the overpotential at 10 mA cm^−2^ for the anisotropic substitution series. Here, a clear correlation of activity with Co^3+^ content (stated in brackets in the legend of Figure 8) of the spinels is observed for both the CAN test and the electrochemical OER. Initial rates for chemical water oxidation decrease with decreasing Co^3+^ content and the overpotentials for the OER increase significantly with decreasing Co^3+^ content. Contrarily, the OER performance of differently prepared α‐MnO_2_ catalysts was found not to scale with the CAN trend in a similar comparison of catalysts with different microstructure and the same composition.^[19a]^ For the OER, charge carrier mobility is of importance and electric conductivity is considered as a major beneficial property for oxide electro‐catalysts. This property is not of superior relevance for the chemical oxygen evolution, as the reaction is taking place at the surface in the presence of a liquid oxidant and no electron transfer through the electrode and back contact must occur. The similar trend for CAN test and electrochemical OER suggests that the activity differences indeed can be traced back to variations in the intrinsic activities of the catalysts and their composition.

**Figure 9:**
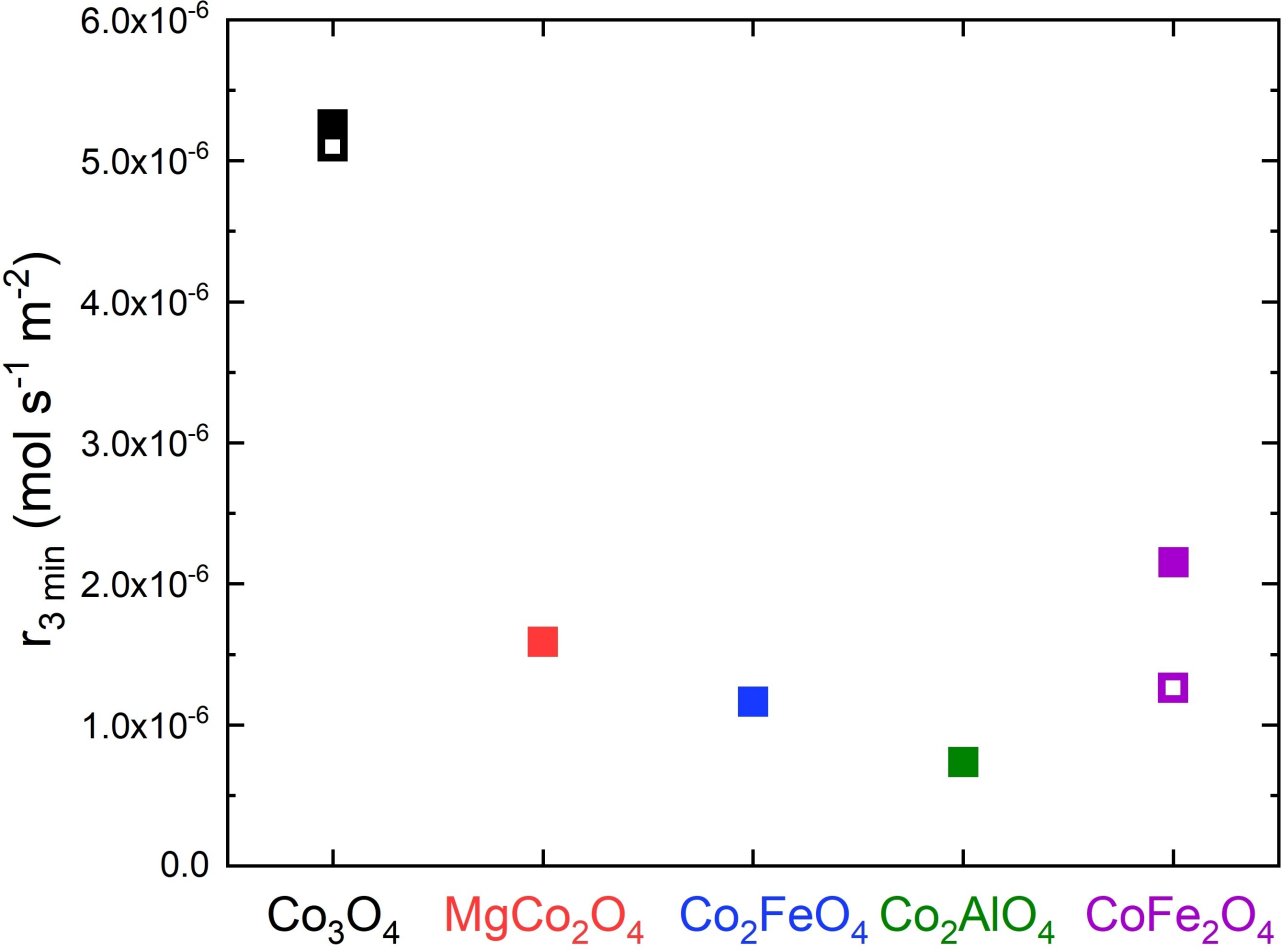
Rates after 3 min for H_2_O_2_ decomposition of the anisotropic cobalt spinel substitution series (closed symbols) and the isotropic samples (open symbols).

With H_2_O_2_ decomposition a reaction is investigated, for which Co^2+^ in octahedral sites is considered to be the active site.^[36]^ Figure 9 shows the rates after 3 min for all samples. The oxygen evolution as a function of time is shown in Figure S18.

As observed for the two reactions presented before, Co_3_O_4_ was the best performing catalyst. The systematic substitution of the redox active cobalt cations with redox inactive magnesium or aluminum leads to a decrease in activity suggesting a trend with the total cobalt content of the mixed oxide. On the other hand, the relevance of Co^2+^ is undeniable when looking at the relative high activity of CoFe_2_O_4_. Considering that ferrite spinels are expected to show a cation ordering described as the inverse spinel structure with iron cations preferably occupying the tetrahedrally coordinated sites according to (Fe^3+^_*t*_)(Co^2+^_*o*_)(Fe^3+^_*o*_)O_4_, this catalyst contains the active octahedral Co^2+^ sites for the H_2_O_2_ decomposition. For the previous reactions, cobalt ferrite showed the lowest activity, therefore in turn indicating the significance of Co^3+^ for the chemical and electrochemical oxygen evolution. Similar to the electrochemical OER, the anisotropic samples with a preferred 111 termination outperform their isotropic counterparts. On one side, this effect is very small for the Co^2+^ and Co^3+^ containing Co_3_O_4_, but the activity of the only Co^2+^ containing cobalt ferrite is boosted significantly with this preferred orientation.

**Figure 10:**
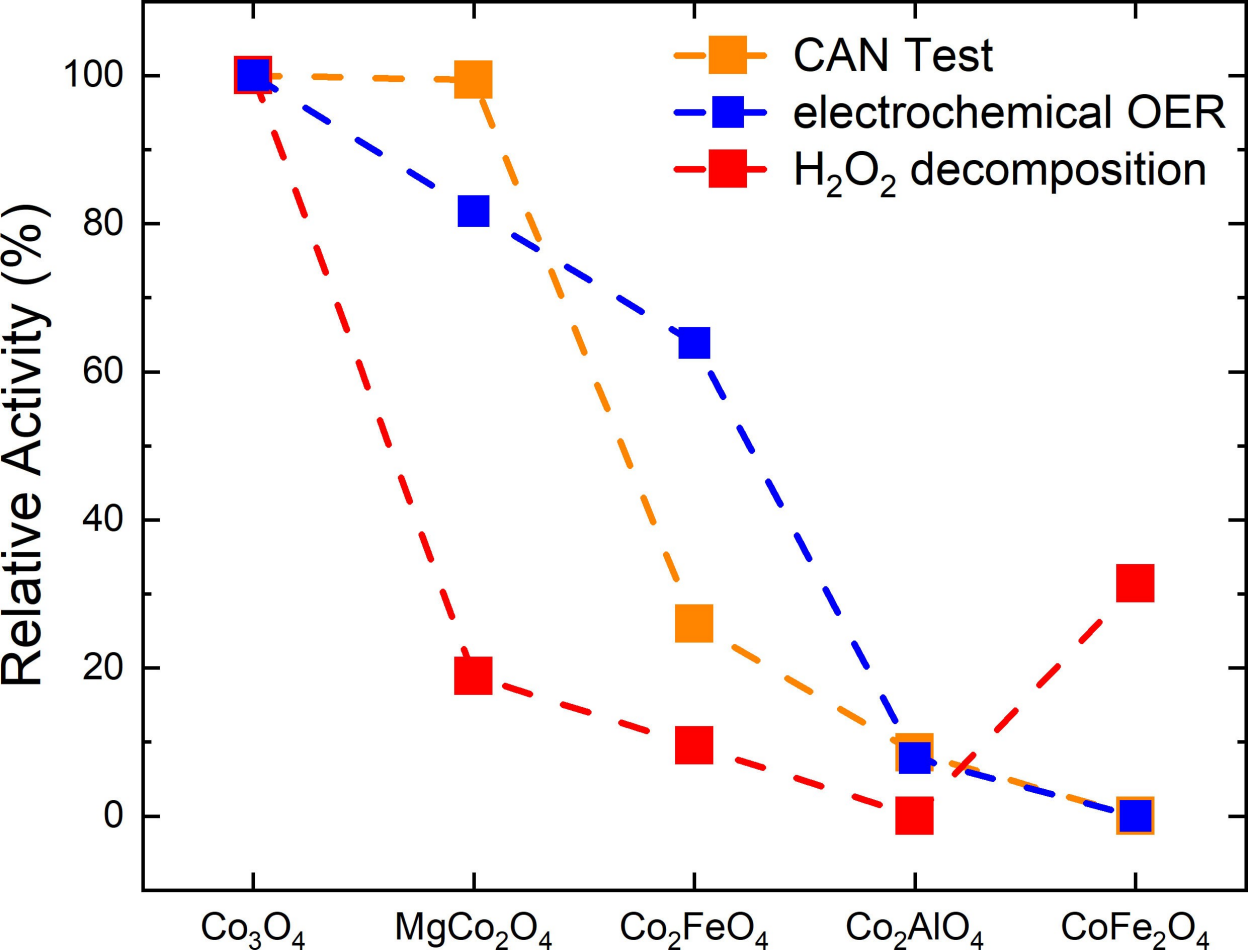
Normalized relative activity for the three investigated reactions. The most active catalyst was set to 100 % performance and the least active to 0 % to highlight the relative differences.

Figure 10 shows the normalized initial rates for chemical water oxidation, H_2_O_2_ decomposition and the overpotential at 10 mA cm^−2^ for electrochemical OER for the anisotropic spinel substitution series. Despite the different nature of all oxygen evolution reactions studied here, the catalyst with the highest cobalt content, Co_3_O_4_, is always identified as the most active catalyst within the anisotropic substitution series highlighting the catalytic role of this element for oxygen evolution. When substituting other cations for cobalt, a loss in activity was observed for all compositions and reactions except for MgCo_2_O_4_ in the CAN test. This sample where Co^2+^ was substituted by Mg^2+^, was the second‐best catalyst underlining the catalytic role of the remaining Co^3+^ for the CAN test and the electrochemical OER with only a little activity loss. In case of the H_2_O_2_ decomposition, a substantial activity loss was observed when going from Co_3_O_4_ to MgCo_2_O_4_, which is consistent with a Co^2+^‐centered reaction as suggested in the literature.^[36]^ Within the samples with two thirds of cobalt cations, there is a consistent and almost linear decreasing trend when going from MgCo_2_O_4_ to Co_2_FeO_4_ and finally to Co_2_AlO_4_ suggesting that the positive effect of Co^3+^ is more important than the promoting effect of the redox‐active iron cation. The latter effect was most prominent when comparing Co_2_FeO_4_ and Co_2_AlO_4_ in electrochemical OER and may be related to a better “hopping” conductivity when Fe‐based redox pairs are present in the spinel structure. On a relative scale, the performance of the Co_2_AlO_4_ catalyst is consistently at the lower end for all three reactions, but the CoFe_2_O_4_ was the worst catalyst in the CAN test and the OER showing again that the promoting effect by iron cannot compensate the lower amount of cobalt and especially the absence of Co^3+^ in this material. However, in the H_2_O_2_ decomposition, CoFe_2_O_4_ shows the second highest activity behind Co_3_O_4_.

The investigation of the activity of different mesostructures showed a higher activity for the preferentially 111 terminated Co_3_O_4_ compared to the isotropic counterpart with no predominant surface termination. With no Co^3+^ in the spinel structure present as it is the case for cobalt ferrite, predominant surface termination does not have a systematic effect on the activity in the herein investigated reactions. Based on this observation, it can be speculated that the beneficial effect of Co^3+^ species is particularly strong if these species reside in a 111 spinel termination. With oxygen ligands, Co^3+^ is expected to be most stable in octahedral low spin configuration (normal spinel structure) rendering the B site termination a promising candidate for the catalytically active surface in spinels or for its precursor. In conclusion, these findings underline the complexity of heterogeneous catalysts and the importance of composition and mesostructure for a given reaction, especially surface termination, and they at the same time highlight the potential of comparative investigations on systematic sample series to tackle this complexity.

## Conclusion

A thoroughly characterized sample series of cobalt‐based spinel oxides was synthesized by co‐precipitation employing the crystalline precursor decomposition approach. The as‐prepared (layered double) hydroxide precursors as well as the desired spinel catalysts were shown to be phase pure and exhibited an anisotropic platelet morphology. The topotactic transformation of the precursors into the spinel catalysts enables a high control over the catalysts mesostructure, especially surface termination, cation distribution and porosity. The cationic composition of the mixed oxides was systematically varied by substitution of Co^2+/3+^ by Mg^2+^, Fe^3+^ and Al^3+^. Furthermore, the cobalt spinel and cobalt ferrite exhibiting an anisotropic platelet‐like morphology, were compared to isotropic Co_3_O_4_ and CoFe_2_O_4_ with more spherical particles. The oxygen evolving CAN test, the electrochemical OER and the decomposition of H_2_O_2_ were used as probe reactions to study the catalytic properties of the spinel samples.

For all reactions Co_3_O_4_ is identified as the most active catalyst within the anisotropic substitution series. All samples containing Co^3+^ show the same trend with substitution. For the two water oxidation reactions, even a semi‐quantitative similarity was observed rendering the CAN test a robust probe reaction for OER for these catalysts. With decreasing amount of Co^3+^ the activity decreases as well. Furthermore, redox active cations, such as iron, enhance the catalytic performance when the same relative Co^3+^ content is considered. For the CAN test and the electrochemical OER Co^3+^ clearly plays a major role for the catalytic reactivity. In contrast Co^2+^ dominates for H_2_O_2_ decomposition, showing a higher relative activity for the only Co^2+^ containing cobalt ferrite. These results underline the general importance of cobalt and of Co^3+^ in particular for the water oxidation reactions and are in agreement with the current literature. The investigation of the activity of different mesostructures showed a higher activity for the 111 terminated Co_3_O_4_ compared to the isotropic counterpart with no predominant surface termination suggesting an important role of Co^3+^ species in this facet, most likely with the B site termination.

These proposals for structure reactivity relationships were enabled by controlled synthesis conditions, thorough characterization, and precise kinetic testing. Further investigations for validation are required and will include operando and in situ techniques to give additional insight into factors governing the dynamics and activity of the solid‐liquid interface. The approach to develop inspiration for such work from composition‐ and mesostructured‐activity correlations can be extended in the future to other heterogeneous oxidation reactions, looking into additional selectivity issues not common to oxygen evolution.

## Experimental Section

**Synthesis**: All syntheses were conducted in an automatic lab reactor system (*OptiMax 1001, Mettler Toledo*). Co‐precipitation of the hydroxide and layered double hydroxide precursors was achieved by simultaneously dosing a metal salt solution and a precipitation agent into the reactor, prefilled with 200 mL of distilled water. The pH was controlled by an InLab Semi‐Micro‐L pH electrode and kept at a fixed value by automatically dosing the precipitation agent. The temperature was kept constant during the whole synthesis procedure. All precipitates were washed by repeatedly dispersing in demineralized water and subsequent centrifugation until the conductivity of the supernatant was below 100 μm S.

**Co_3_O_4_**: For the Co(OH)_2_ precursor 125 g of a 0.8 M Co(NO_3_)_2_x6H_2_O (≥98 %, Carl Roth) solution were dosed continuously over 1 h at pH 8.5 and 50 °C with 0.6 M NaOH (98.5 %, VWR) as precipitation agent. Following precipitation, the material was aged for 1 h without pH control at 50 °C. During the reaction, the reactor was continuously purged with N_2_ and stirred at 350 rpm. After cooling to room temperature, the precipitate was washed with water several times and dried at room temperature in vacuum. Subsequently the dried precursor was calcined at 400 °C (2 K min^−1^) for 3 h in a muffle furnace (*Nabertherm LE 6/11/B150*).

**MgCo_2_O_4_**: 125 g of a metal salt solution containing 0.533 M Co(NO_3_)_2_x9H_2_O and 0.266 M Mg(NO_3_)_2_x9H_2_O (99 %, Fisher Scientific) were dosed in 1 h at pH 11.0 and 50 °C with a 1.0 M NaOH as precipitation agent. Following precipitation, the material was aged for 1 h without pH control at 50 °C. During the reaction, the reactor was continuously purged with N_2_ and stirred at 350 rpm. After cooling to room temperature, the precipitated Mg_1/3_Co_2/3_(OH)_2_ was washed with water several times and dried overnight at 80 °C in static air. Subsequently the dried precursor was calcined at 400 °C (2 K min^−1^) for 3 h in a muffle furnace.

**Co_2_FeO_4_**: For precipitation of a Co^2+^Fe^3+^ LDH precursor 125 g of a metal salt solution containing 0.533 M Co(NO_3_)_2_x9H_2_O and 0.266 M Fe(NO_3_)_3_x6H_2_O (≥98 %, Sigma Aldrich) were dosed in 1 h at pH 8.5 and 50 °C with a precipitation agent containing 0.6 M NaOH and 0.09 M Na_2_CO_3_ (≥99.5 %, Carl Roth). Following precipitation, the material was aged for 1 h without pH control at 50 °C. During the reaction, the reactor was continuously stirred at 350 rpm. After cooling to room temperature, the precipitated Mg_1/3_Co_2/3_(OH)_2_ was washed with water several times and dried overnight at 80 °C in static air. Subsequently the dried precursor was calcined at 400 °C (2 K min^−1^) for 3 h in a muffle furnace.

**Co_2_AlO_4_**: For precipitation of a Co^2+^Al^3+^ LDH precursor 125 g of a metal salt solution containing 0.533 M Co(NO_3_)_2_x9H_2_O and 0.266 M Al(NO_3_)_3_x6H_2_O (≥98 %, Carl Roth) were dosed in 1 h at pH 10.0 and 50 °C with a precipitation agent containing 1.0 M NaOH and 1.2 M Na_2_CO_3_. Following precipitation, the material was aged for 1 h without pH control at 50 °C. During the reaction, the reactor was continuously stirred at 350 rpm. After cooling to room temperature, the precipitated Mg_1/3_Co_2/3_(OH)_2_ was washed with water several times and dried overnight at 80 °C in static air. Subsequently the dried precursor was calcined at 400 °C (2 K min^−1^) for 3 h in a muffle furnace.

**CoFe_2_O_4_**: For precipitation of a Co^2+^Fe^2+^Fe^3+^ LDH precursor 125 g of a metal salt solution containing 0.133 M CoCl_2_x6H_2_O (99.9 %, Alfa Aesar), 0.133 M FeCl_2_x4H_2_O (>99 % Acros Organics) and 0.133 M FeCl_3_x6H_2_O (≥99 %, Carl Roth) were dosed in 1 h at pH 8.5 and 10 °C with precipitation agent containing 0.6 M NaOH and 0.09 M Na_2_CO_3_. Following precipitation, the material was aged for 24 h without pH control at 10 °C. During the reaction, the reactor was continuously purged with N_2_ and stirred at 350 rpm. After cooling to room temperature, the precipitated Co^2+^Fe^2+^Fe^3+^ LDH was washed with water several times and dried at room temperature in vacuum. Subsequently the dried LDH precursor was calcined at 600 °C (2 K min^−1^) for 3 h in a muffle furnace.

**Isotropic Co_3_O_4_**: Isotropic Co_3_O_4_ was synthesized by thermal treatment of a precipitated hydroxy carbonate precursor. The automatic lab reactor system described above was prefilled with 200 mL of a 0.5 M Co(NO_3_)_2_x6H_2_O solution. The prefilled solution was stirred at 350 rpm, held at 25 °C and a pH of 9 was set with a 1 M Na_2_CO_3_ solution. After aging for one hour without pH control, the precipitate was washed and dried overnight in static air at 100 °C. The dried precursor was calcined at 500 °C (2 K min^−1^) for 3 h in a muffle furnace.

**Isotropic CoFe_2_O_4_**: Isotropic CoFe_2_O_4_ was directly precipitated. Therefor a 125 g of a metal salt solution containing 0.233 M Co(NO_3_)_2_x9H_2_O and 0.566 M Fe(NO_3_)_3_x6H_2_O were dosed into the prefilled (200 mL distilled H_2_O) reactor over the course of 1 h. A constant pH of 10 was kept with a 1.5 M NaOH solution and the temperature was held at 10 °C. Following precipitation, the material was aged for 1 h without pH control at 10 °C. During the reaction, the reactor was continuously purged with N_2_ and stirred at 350 rpm. After cooling to room temperature, the precipitated CoFe_2_O_4_ was washed with water several times and dried at 80 °C in static air. Subsequently the dried precipitate was calcined at 500 °C (2 K min^−1^) for 3 h in a muffle furnace.

**Characterization**: Powder X‐ray diffraction (PXRD) was performed with a Bruker D8 Advance diffractometer with a Cu X‐ray source in Bragg‐Brentano geometry, using a LynxEye XE‐T detector. The samples were dispersed in ethanol on a PMMA sample holder and diffraction patterns were recorded in the angular range from 5 ° to 90 ° 2θ with a step size of 0.01 ° and a counting time of 1.5 s. During the measurement, the sample holder was slowly rotated. For showing phase purity, determination of lattice parameters of the as‐prepared spinels Rietveld refinement was performed with the Bruker software TOPAS.

Scanning electron microscopy (SEM) was conducted with an Apreo S LoVac (Thermo Fisher Scientific). Prior to the measurements, the samples were sputtered with Pt/Au.

The ratio of the incorporated metal cations in the spinel catalysts was determined by X‐ray fluorescence using a Bruker S8 TIGER Series (4 kW).

Thermogravimetric analysis (TGA) of the as‐prepared precursors was carried out with a NETZSCH STA 449f F3 Jupiter (NETSCH GmbH, Germany). The mass loss was recorded as a function of the temperature with a linear heating rate of β=5 K min^−1^ in a temperature range from 30 °C to 1000 °C and a gas stream of O_2_ (21 mL min^−1^) and Ar (79 mL min^−1^).

BET surface areas were measured using N_2_ physisorption at 77 K with a NOVA3000 (Quantachrome GmbH, Germany). Prior to the measurements, the samples were degassed at 80 °C in vacuum for 2 h. Pore volume and the pore size distribution were determined by applying the BJH method.

Mössbauer spectra were recorded on powder samples in standard transmission geometry, using a ^57^Co radiation source mounted on a WissEl driving unit operating in constant acceleration mode. A liquid helium bath cryostat was utilized to attain low temperatures and high fields, containing a superconducting solenoid in split‐pair geometry. A homogenous magnetic field of 5 T was applied at the sample position, with the field orientation parallel to the γ‐ray propagation direction (in‐axis).

Magnetic properties were analyzed with the vibrating sample magnetometer (VSM) option of a Quantum Design PPMS DynaCool. The field dependent properties were characterized via M(H) loops recorded at 4.3 K and 300 K at magnetic fields up to 9 T, while temperature dependent properties were recorded via M(T) sweeps between 5 K and 400 K using the zero field cooled‐field cooled (ZFC‐FC) regime at an applied field of 1 T.

TEM micrographs were recorded with a JEOL 2200FS with a probe‐side Cs‐corrector operated at 200 kV acceleration voltage. Selected area electron diffraction images were taken at a camera length of 30 cm.

XPS measurements were performed on a VersaProbe II micro‐focus X‐ray photoelectron spectrometer (UlvacPhi) using monochromatic Al−Kα light at 1486.6 eV photon energy. Charging effects were compensated using a dual‐beam neutralizing approach using electrons and slow moving argon ions.

**Catalysis**: Chemical water oxidation was performed using Ce^4+^ as oxidizing agent. During the oxidation process of water to oxygen the single‐electron oxidant Ce^4+^ is reduced to Ce^3+^ (E^0^(Ce^3+^/Ce^4+^)=1.72 V vs. NHE at pH=0) [Eq. 1].

(1) $${4Ce}^{4+}+2H_{2}O\overset{spinel\;catalyst}{\underset{}{\to }}{4H}^{+}+O_{2}+{4Ce}^{3+}$$

For determination of the initial rates for chemical water oxidation 100 mg of catalyst were dispersed in 45 mL water. The setup was purged with Ar (50 mL min^−1^) to ensure an oxygen free environment. 5 mL of a (NH_4_)_2_Ce(NO_3_)_6_ solution (2.5 mol L^−1^) were added to the dispersion and the evolving oxygen was detected by an oxygen analyzer (EC900, Systech Illinios) over 2 h. For each catalyst twofold measurements were performed.

**Electrochemical measurements**: The determination of the OER activity and the C_DL_ were carried out in a 3‐electrode electrochemical cell using an AUTOLAB PGSTAT 204'' potentiostat and a Metrohm rotator. The working electrode consisted of drop‐casted catalyst ink on a glassy carbon (GC) disk, a double junction Ag/AgCl/3 M KCl/1 M KOH electrode was used as reference electrode, and a platinum mesh which was separated by a frit acted as the counter electrode. The catalyst ink was prepared by dispersing 1 mg of catalyst ink in 98 μL water, 98 μL ethanol and 4 μL Nafion (∼5 % (w/v)) which was used as binder. The GC electrode was polished using 0.05 μm alumina paste until a mirror‐like finish was achieved. 4.8 μL of the catalyst ink was drop‐casted on the GC electrode to acquire a final loading of 210 μg/cm^2^. Argon saturated 1 M KOH was used as the electrolyte. Prior to use, the electrolyte was purified by removing metal impurities using a chelating ion resin (Chelex 100, Bio‐Rad). A small stream of argon was flushed continuously during the measurement over the electrolyte to maintain gas saturation. The electrochemical activity was measured using linear sweep voltammetry (LSV). The LSVs were recorded at a scan rate of 0.005 V/s at a rotation speed of 1600 rpm. Each catalyst was measured in triplicate. Each measurement included a galvanostatic impedance measurement at 0 A, which was later used to correct for the uncompensated solution resistance. The C_DL_ measurements were done by potentiodynamic cycling in the non‐faradaic region of the voltammogram at different scan rates of 0.01, 0.05, 0.1, 0.15, 0.20 V/s. The anodic and cathodic currents were extracted, and the average was plotted against the respective scan rate. The slope of the linear regression was used to determine C_DL_. All potentials were converted to RHE using the following Equation 2.

(2) $$E{}_{\mathrm{RHE}}=E{}_{\mathrm{Ag}/\mathrm{AgCl}/\mathrm{KCl}}+0.207+0.059{}^{*}\mathrm{pH}$$

H_2_O_2_ decomposition was carried out in a peroxide decomposition set up (Gassmess‐5). 10 mg catalyst were dispersed in 30 mL acetonitrile. The solution was kept at 30 °C and stirred at 600 rpm. 80 μL H_2_O_2_ (30 wt.%) were added and the gas volume measurement was started immediately. The decomposition reaction was run for 30 min.

## Conflict of interest

The authors declare no conflict of interest.

## Supporting information

As a service to our authors and readers, this journal provides supporting information supplied by the authors. Such materials are peer reviewed and may be re‐organized for online delivery, but are not copy‐edited or typeset. Technical support issues arising from supporting information (other than missing files) should be addressed to the authors.

Supporting Information
