# Supplementary material for: The Roles of Composition and Mesostructure of Cobalt‐Based Spinel Catalysts in Oxygen Evolution Reactions
Source: Chemistry. 2021 Oct 22;27(68):17038–48. doi: 10.1002/chem.202102400 (PMC9298119; doi:10.1002/chem.202102400)
Supplement: Supplementary file 1 — Supporting Information [file CHEM-27-17038-s001.pdf]

Chemistry–A European Journal

Supporting Information

The Roles of Composition and Mesostructure of Cobalt-Based Spinel Catalysts in Oxygen Evolution Reactions

Anna Rabe, Julia Büker, Soma Salamon, Adarsh Koul, Ulrich Hagemann, Joachim Landers, Klaus Friedel Ortega, Baoxiang Peng, Martin Muhler, Heiko Wende, Wolfgang Schuhmann, and Malte Behrens*

As reproducibility is of pivotal importance in catalyst synthesis, this aspect is being highlighted for the magnesium cobalt hydroxide precursor and the resulting spinel catalyst. Figure S 1 to Figure S 3 display the synthesis protocols, PXRD patterns and BET analysis to prove batch to batch reproducibility.

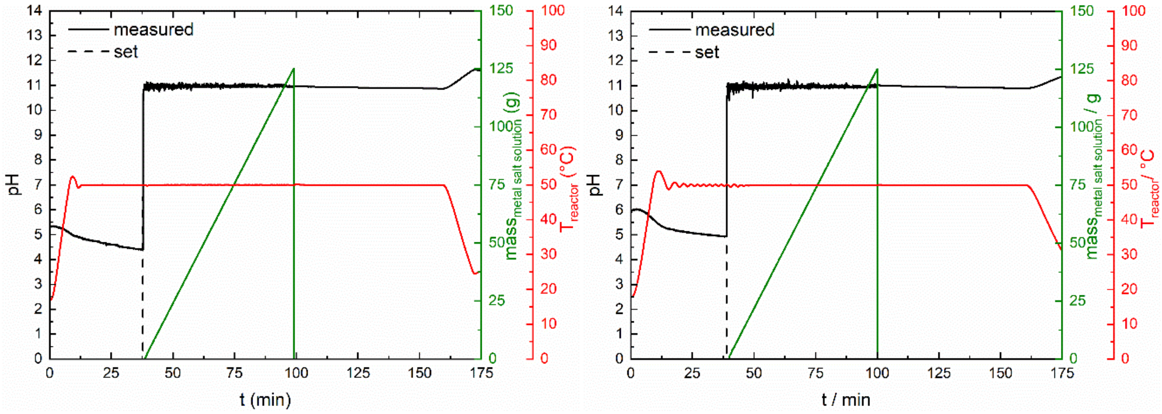

Figure S 1. Synthesis protocols of two different batches $Mg^{2+}_{1/3}Co^{2+}_{2/3}(OH)_2$. pH, dosing speed and temperature are perfectly controlled, therefore ensuring reproducible catalyst properties.

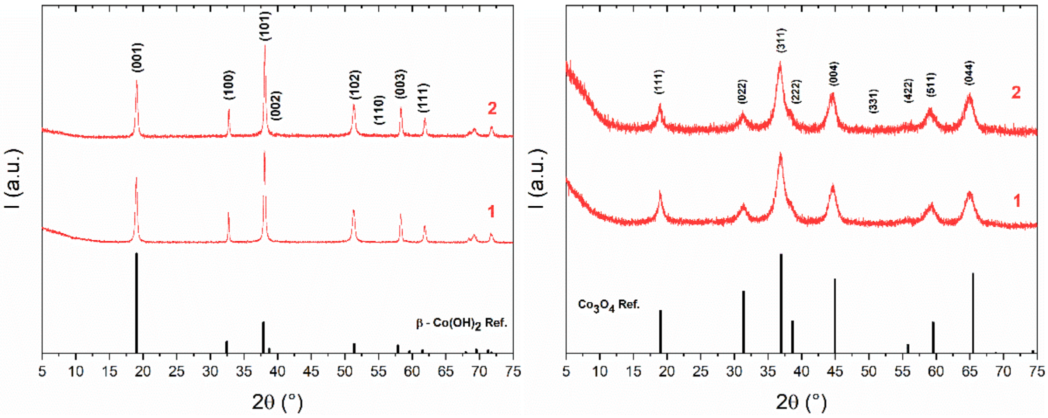

Figure S 2: PXRD patterns of the as-prepared $Mg^{2+}_{1/3}Co^{2+}_{2/3}(OH)_2$precursors (left) and $MgCo_2O_4$ catalysts after calcination at 400 °C (right).

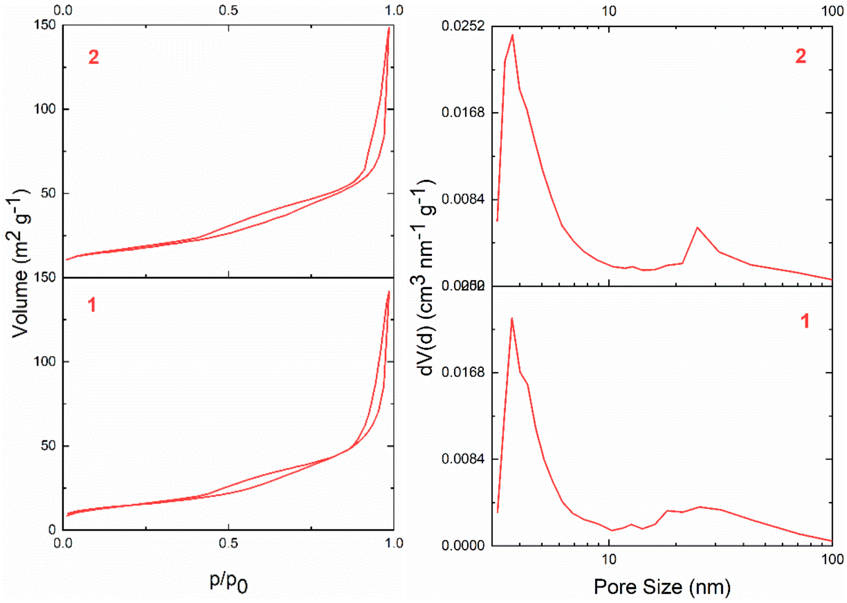

Figure S 3: Adsorption desorption isotherms (left) and pore size distributions (right) of two different batches of $MgCo_2O_4$. The surface area was determined to 55 m^2g^{-1}± 6 m^2g^{-1}.

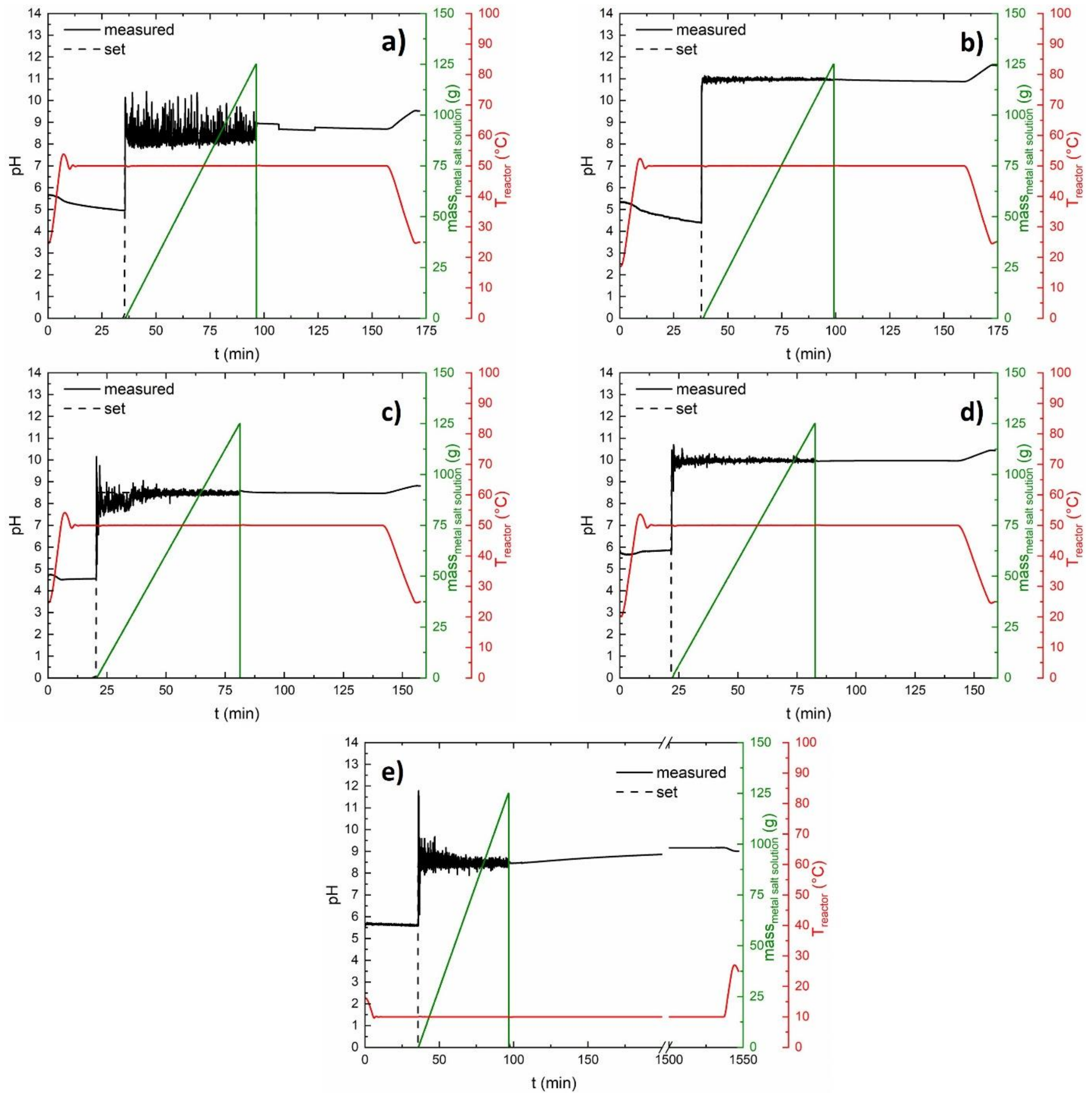

Figure S 4. Synthesis protocols of the precursor precipitation and aging. a) $Co^{2+}(OH)_2$, b) $Mg^{2+}_{1/3}Co^{2+}_{2/3}(OH)_2$, c) $Co^{2+}_{2/3}Fe^{3+}_{1/3}$ LDH, d) $Co^{2+}_{2/3}Al^{3+}_{1/3}$ LDH, e) $Fe^{2+}_{1/3}Co^{2+}_{1/3}Fe^{3+}_{1/3}$ LDH.

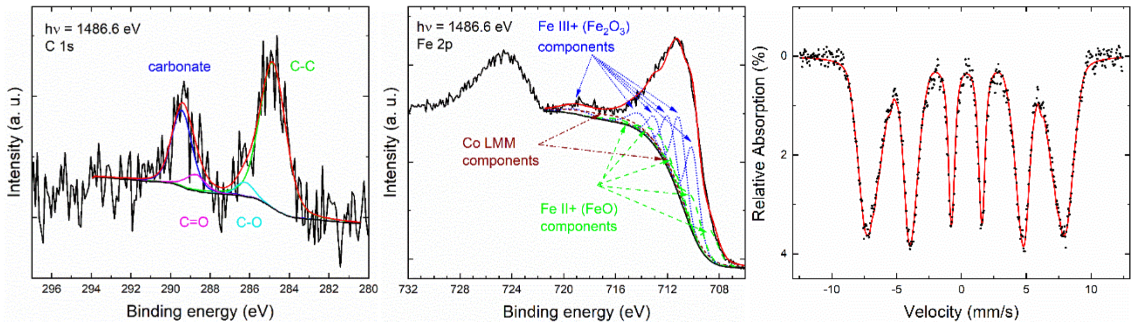

Figure S 5: C 1s (left), Fe 2p, (middle) XP spectra and Mössbauer spectra (right) of the $Fe^{2+}_{1/3}Co^{2+}_{1/3}Fe^{3+}_{1/3}$ LDH precursor.

As Fe^{2+} is easily oxidized to Fe^{3+} in air, the actual existence of Fe^{2+} has to be proven. For this reason, XPS and Mössbauer spectroscopy were performed. XPS showed an amount of 33±10 % Fe^{2+} of the overall Fe content and a total Fe/Co ratio of 1.66±0.15, agreeing with the determined 1.65 ratio from

AAS. Furthermore, XPS showed minor amounts of carbon, underlining the claim of "organic free" synthesis. The expected interlayer carbonate is clearly visible in the C 1s spectrum.

Mössbauer spectra were recorded at low temperature (4.3 K) and high magnetic field (5 T) parallel to the γ-ray propagation direction. For the $Fe^{2+}{}_{1/3}Co^{2+}{}_{1/3}Fe^{3+}{}_{1/3}$ LDH precursor material, the line intensity ratio A_{23} close to 2 reveals mainly antiferromagnetic behavior with random spin orientation, as it would be expected for LDH-based materials.[1] As a result, the evaluation of the experimental data points was achieved by means of an equidistant distribution of magnetic hyperfine fields, as no individual subspectra corresponding to different crystallographic sites could be discerned. With this method, we obtain a mean magnetic hyperfine field of ca. 46 T, with an isomer shift of 0.48 mm/s indicating that we are mainly dealing with Fe^{3+} contributions. This is presumably caused by the extended measurement time required for Mössbauer spectroscopy, and the associated oxidation of the originally present Fe^{2+} fraction, which was verified in XPS measurements. The sample preparation for XPS is comparatively fast and yet not the expected amount of 50 % of Fe^{2+} is found. However, the contribution of the measured Mössbauer spectrum is predominantly from one phase and the X-ray diffraction pattern did not show any sign of a secondary phase. In our recently published paper, a premature spinel by-phase was detected by XRD and Mössbauer spectroscopy.[2] The synthesis, washing and drying procedure in the present work was improved and prevented this premature oxidation and consequential by-phase formation. Even though oxidation occurs during sample preparation and measurement of the X-ray diffraction data, the phase purity is potentially explained by a green rust - mössbauerite related situation.[3] Fe^{2+} oxidizes to Fe^{3+}, but through deprotonation of the hydroxyl groups the resulting additional excess charge in the brucite-like layers is compensated and the structure is stable.

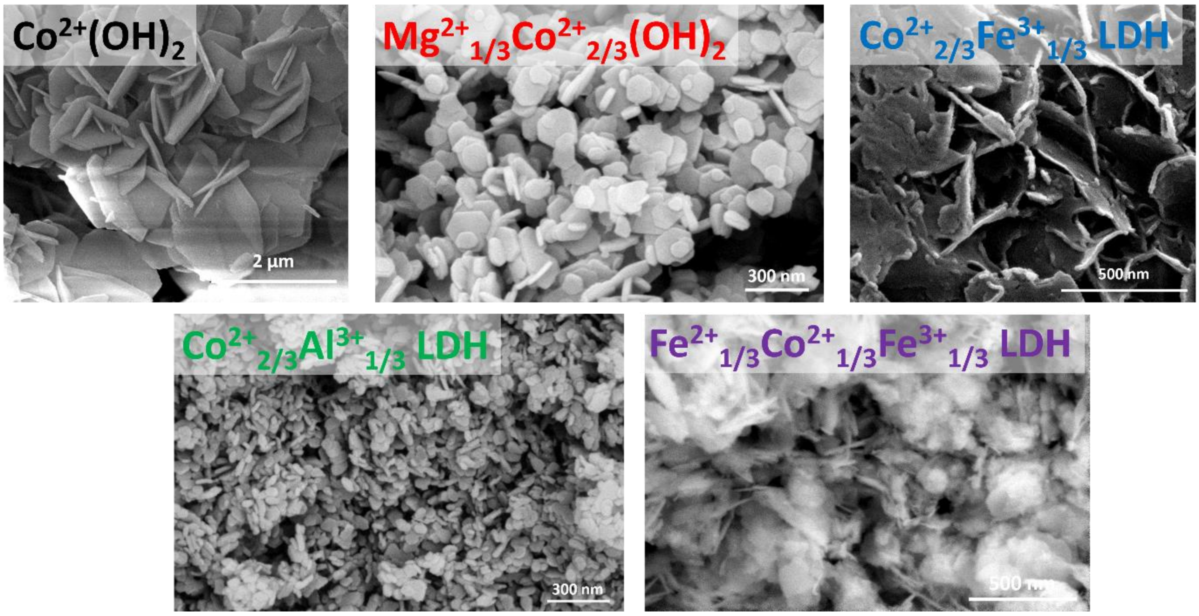

Figure S 6. SEM micrographs of the as-prepared hydroxide and layered double hydroxide precursors.

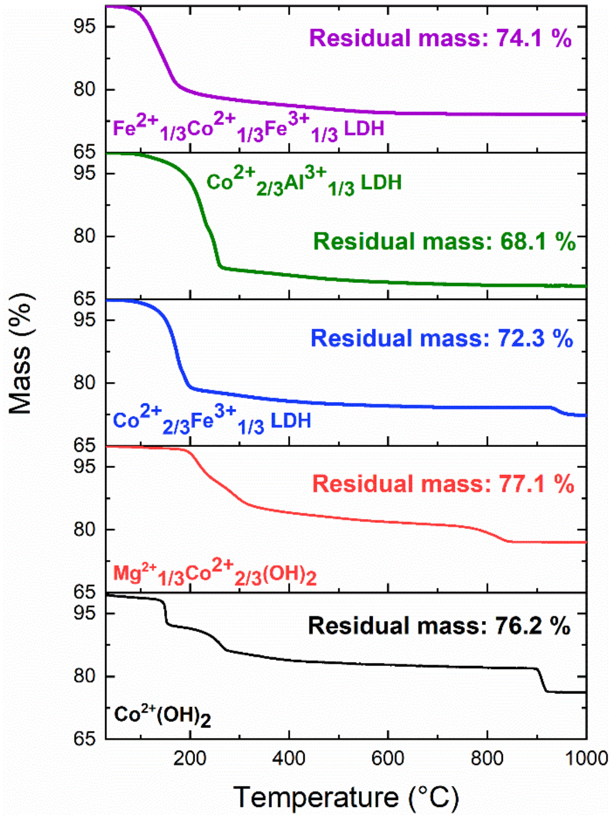

Figure S 7: Relative mass loss as a function of temperature and residual mass for the as-prepared anisotropic precursors.

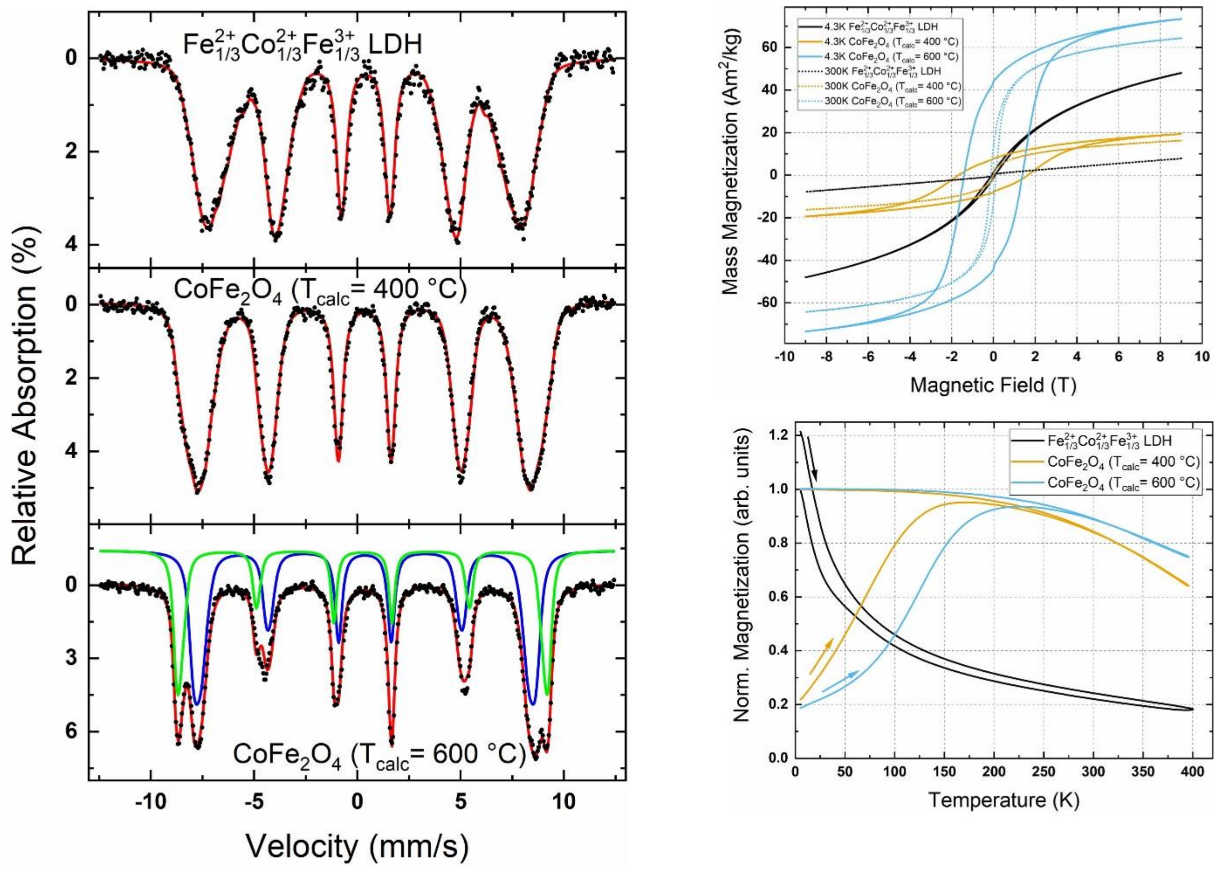

Figure S 8: (a) Mössbauer spectra recorded at 4.3 K and 5 T, M(H) (b) and M(T) (c) measurements of as-prepared layered double hydroxide precursor and samples calcinated at 400°C and 600°C.

In order to obtain a more detailed view on potential structural changes between precursor, resulting product, and possible by-phases, Mössbauer spectra were also recorded for a sample calcined at 400 °C and 600 °C. The results for the $Fe^{2+}_{1/3}Co^{2+}_{1/3}Fe^{3+}_{1/3}$ LDH precursor material are discussed above.

Some changes can be observed for the sample heated to 400 °C, such as the increase of the magnetic hyperfine field to ca. 50 T, pointing towards a general phase change of the material, while the isomer shift is nearly unchanged. The magnetic hyperfine field distribution shows first signs of two spectral contributions, which, together with the reduction in A_{23} to ca. 1.8, representing beginning ferrimagnetic in-field alignment, indicates the starting formation of spinel material.

The most dramatic change takes place after heating to 600 °C, with the resulting spectrum revealing two clearly separated sextet subspectra. Such a spectral structure is typical for ferrimagnetic spinel systems, making it possible to calculate the degree of inversion from the relative spectral areas, as these are proportional to the number of Fe ions on the tetrahedral and octahedral sites. We obtain an inversion parameter of 0.76(3), with this material thus being closer to the inverse (1) than the regular spinel (0), as one would expect for phase pure $CoFe_2O_4$.[5] The mean spin canting angle, derived from A_{23}, decreased to ca. 40°, which is still substantial, usually caused by the high magnetocrystalline anisotropy and canted surface spins. Latter could here be connected to the enhanced surface fraction and the strongly porous nature of the material.

As Mössbauer spectra displayed a considerable change in magnetic structure, the formation of spinel material from the LDH precursor was also investigated by magnetometry. The M(H) sweep of the LDH precursor sample displays a quasi-paramagnetic behavior at room temperature, while a slow rise in magnetization is seen at 4.3 K, indicative of the strongly canted state and resulting lack of full saturation. A very minute saturation effect is observable at very small fields at 300 K, which could represent a miniscule parasitic phase. After heating to 400 °C, the 9 T magnetization at 4.3 K has decreased from 48 Am^2/kg to ca. 19 Am^2/kg, while the sample now also shows a significant remanence and coercive field that were almost completely absent in the the untreated sample. The most dramatic change takes place after heating to 600 °C, where we observe a very broad hysteresis curve at 4.3 K, with a high magnetization of ca. 73 Am^2/kg at 9 T. This value is only slightly below the literature value of 80 Am^2/kg[6] for pure, well-ordered $CoFe_2O_4$ nanoparticles, which can be explained by the still substantial degree of spin canting deduced from the 5 T Mössbauer spectrum for this sample. We can therefore assume that this slightly lowered magnetization is caused by unordered surface spins, and not by an impurity of the material at hand. Regarding the M(T) sweeps, we can of course observe the same changes in magnetization, but as the materials are not in saturation at the applied field of 1 T, we chose to normalize the curves to the end points of the FC branches. Thanks to this, we can directly compare the qualitative temperature dependent progression of each sample's magnetization. We can observe that the LDH precursor does not display any distinct features, with the magnetization continuously dropping towards higher temperatures after attaining the paramagnetic state. In contrast, both the 400 °C and the 600 °C heated sample display typical relaxation behavior known from magnetic nanoparticles, with the inflection points of ca. 86 K for the 400 °C and 130 K for the 600 °C sample clearly showing a rise in blocking temperatures.

In conclusion the magnetic investigations underline the phase purity of the sample calcined at 600 °C, indicating a possible minor by-phase or incomplete transformation for the sample calcined at 400 °C.

Isotropic Co_3O_4 and $CoFe_2O_4$ was synthesized to further investigate the impact of morphology on the catalytic activity. In Figure S9 to S12 characterization data of the before mentioned can be found.

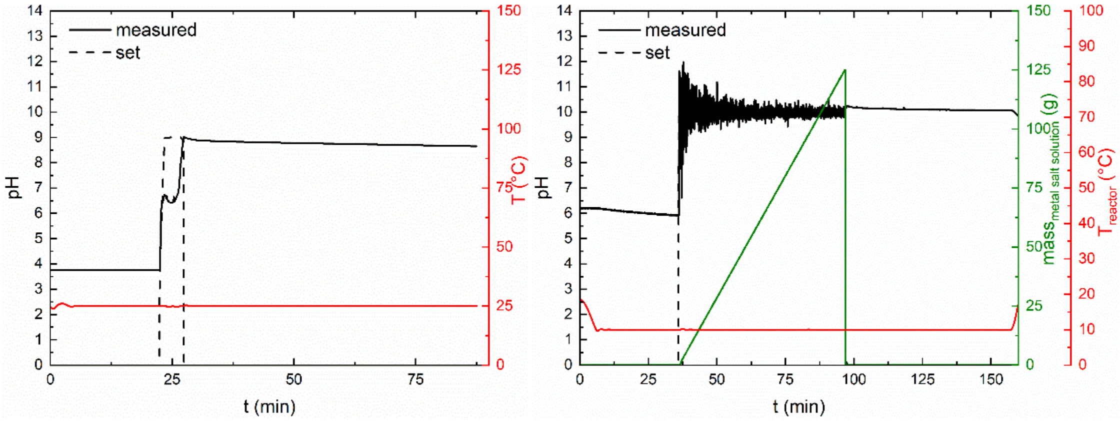

Figure S 9. Synthesis protocols of iso-Co_3O_4 (left) and iso-$CoFe_2O_4$(right).

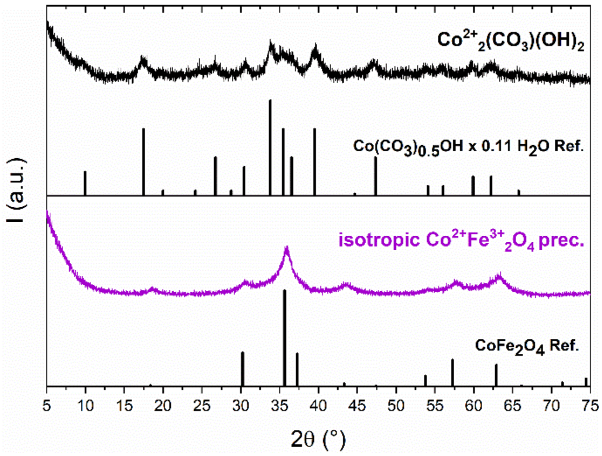

Figure S 10. As-prepared precursor for isotropic Co_3O_4 and $CoFe_2O_4$.

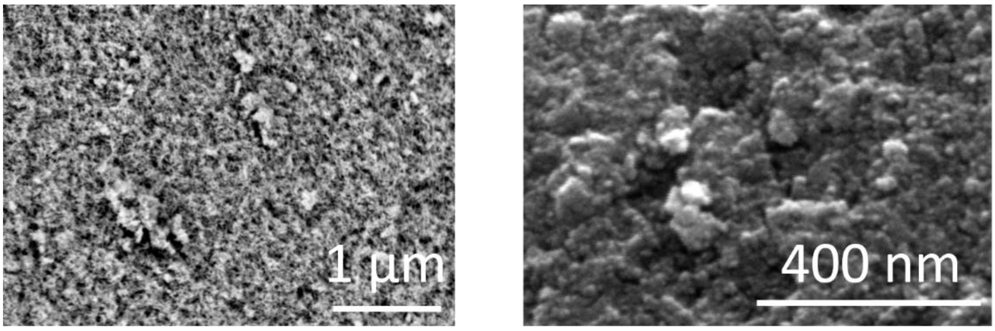

Figure S 11. SEM micrographs of the as-prepared cobalt hydroxy carbonate precursor (left) and directly precipitated $CoFe_2O_4$.

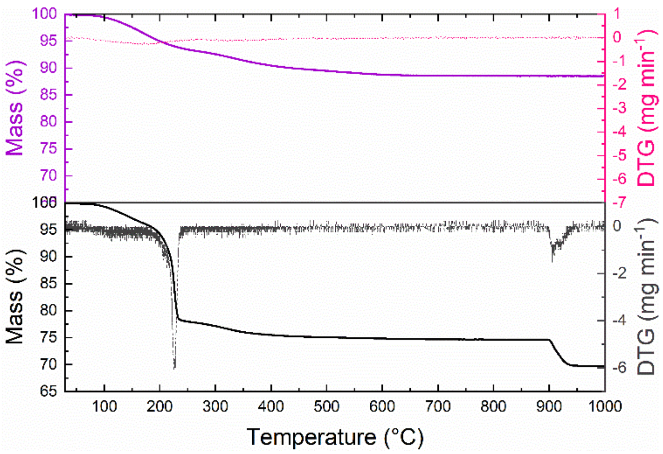

Figure S 12. Thermogravimetric analysis of the precursors for isotropic Co_3O_4 (bottom) and $CoFe_2O_4$ (top).

Table S 1. Rietveld refinement parameters for the anisotropic substitution series and isotropic cobalt spinel and cobalt ferrite.

Sample	R_{exp}	R_{wp}	R_p	R_{exp}'	R_{wp}'	R_p'
Co_3O_4	6.51	7.24	5.76	11.83	13.16	12.37
$MgCo_2O_4$	5.45	6.46	5.12	12.04	14.29	12.34
Co_2FeO_4	6.21	7.22	5.71	9.04	10.51	8.91
Co_2AlO_4	5.59	5.86	4.64	9.49	9.94	8.5
$CoFe_2O_4$	12.56	14.96	11.54	12.65	15.07	12.2
iso-Co_3O_4	6.1	7.17	5.68	10.22	12	10.86
iso-$CoFe_2O_4$	10.44	11.58	8.85	11.57	12.83	10.24

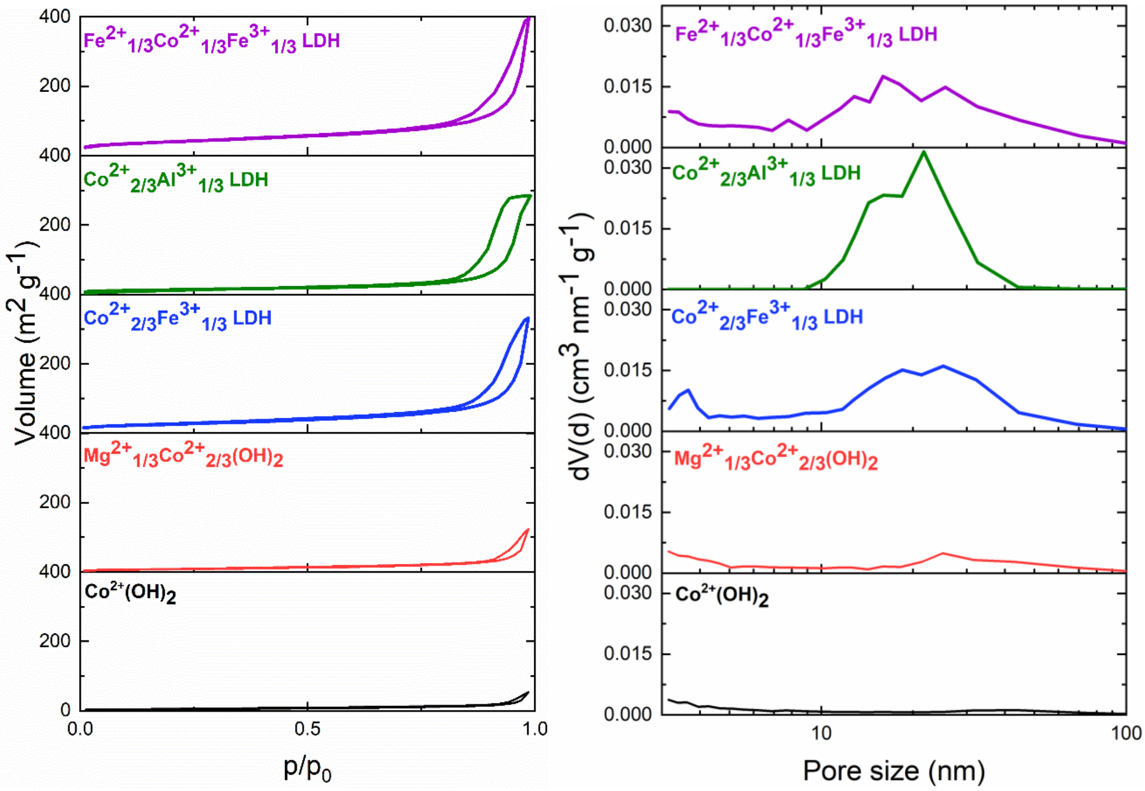

Figure S 13. Adsorption desorption isotherms and pore size distributions of the as-prepared (layered double) hydroxide precursors of the anisotropic spinel substitution series.

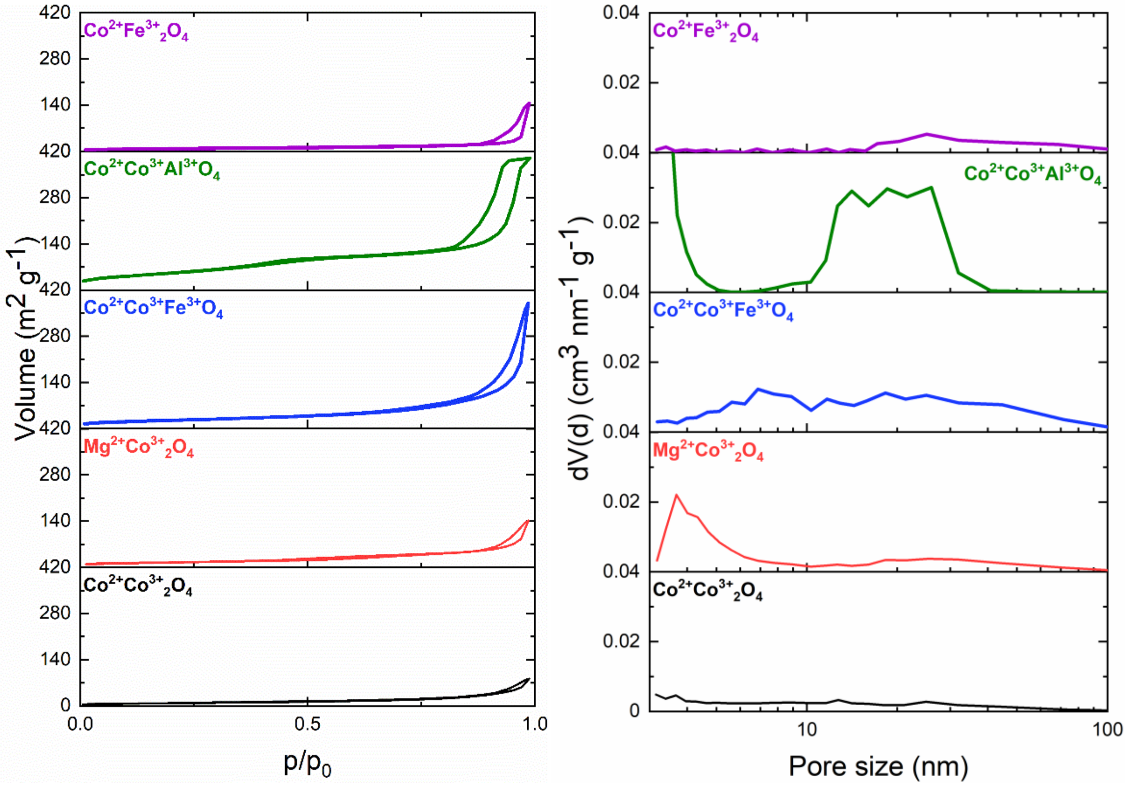

Figure S 14. Adsorption desorption isotherms of the anisotropic spinel substitution series.

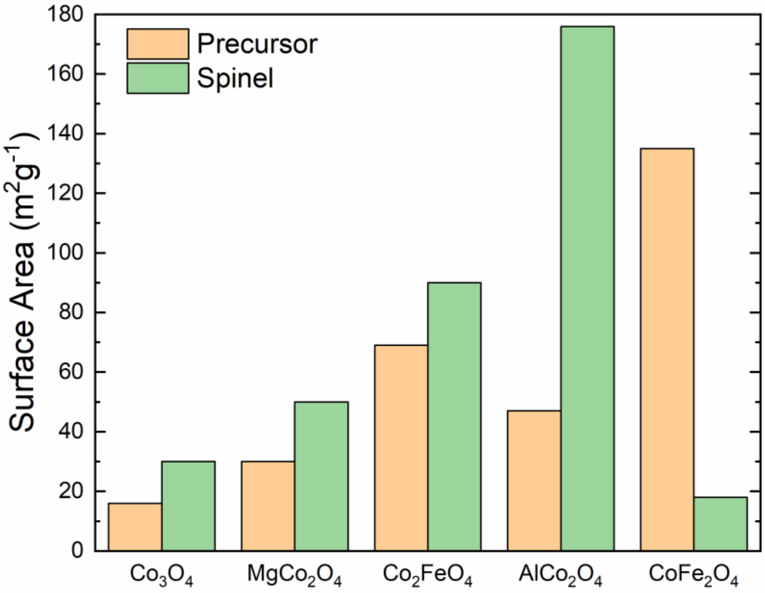

Figure S 15. Comparison of surface areas of as-prepared precursors and calcined spinels. For the cobalt ferrite a decrease in surface area is observed because of the higher calcination temperature.

To analyze the preferred surface termination of the anisotropic samples and to show a more statistical facet distribution for the isotropic analogs, TEM measurements and electron diffraction were performed. Due to the intergrown nature of the anisotropic particles, it was not possible to achieve electron diffraction patterns of a single platelet in most cases. The aggregates make a surface termination determination nearly impossible. Nevertheless, a 111 surface termination is considered for all samples of the anisotropic substitution series. For Co_3O_4 several reports can be found in literature, confirming the topotactic spinel formation.[7] The related dehydroxylation of $Mg(OH)_2$ to MgO has been investigated as well.[8] For $CoFe_2O_4$ we recently showed the same transformation and for Co_2FeO_4 it was shown in the present work (See. Figure 5 in the main text).[2] The 111 planes of the spinel and the 001 planes of the brucite type hydroxides exhibit a very similar d-spacing and therefore transformation occurs along them. This topotactic transformation leads to the preservation of the morphology. As all anisotropic spinels clearly exhibit the hexagonal platelet morphology of their (layered double) hydroxide precursor, this is additional prove for the suggested predominant surface termination.

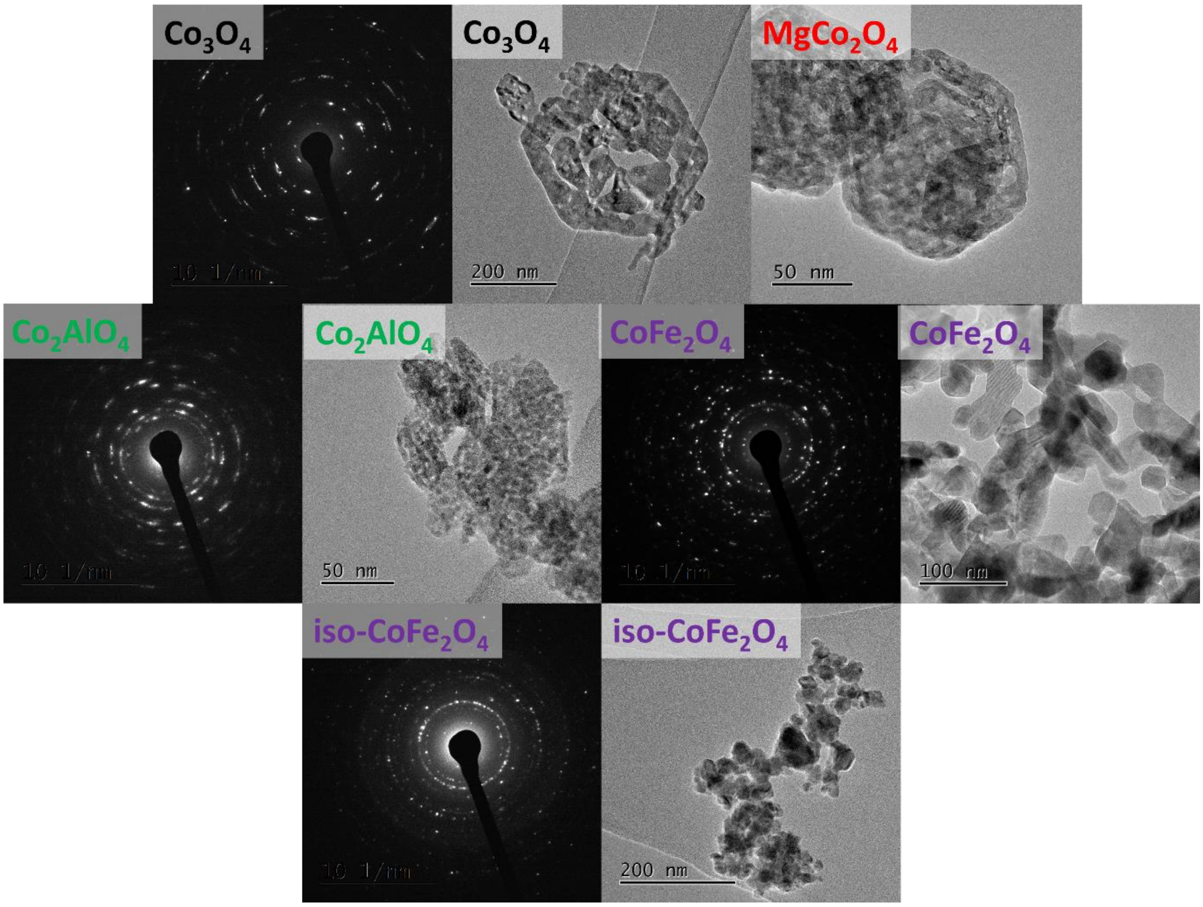

Figure S 16: TEM images and electron diffraction of anisotropic Co_3O_4, $MgCo_2O_4$, Co_2AlO_4, $CoFe_2O_4$ and isotropic $CoFe_2O_4$.

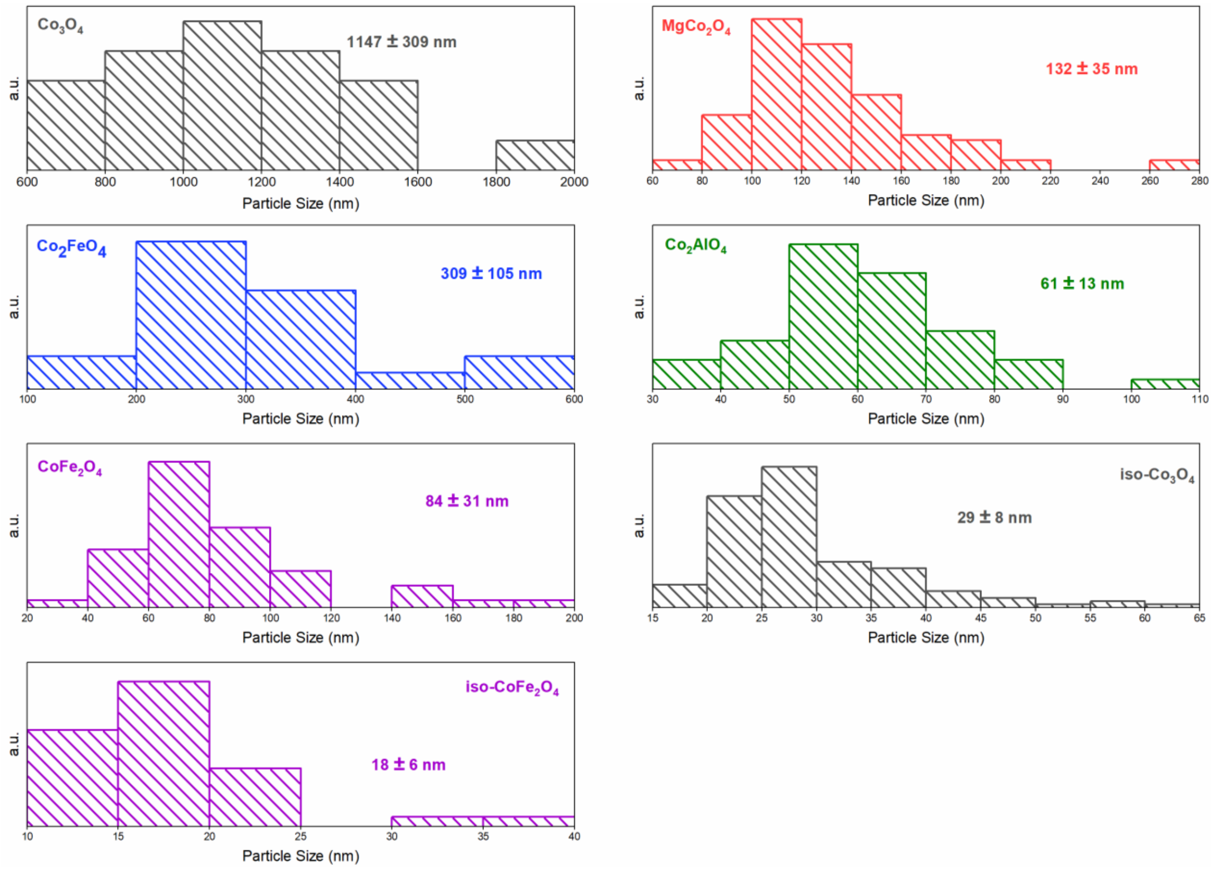

Figure S 17: Particle size histograms of the anisotropic substitution series and the isotropic Co_3O_4 and $CoFe_2O_4$. Particles sizes for the anisotropic substitution series were obtained from SEM micrographs and for the isotropic spinels from TEM images.

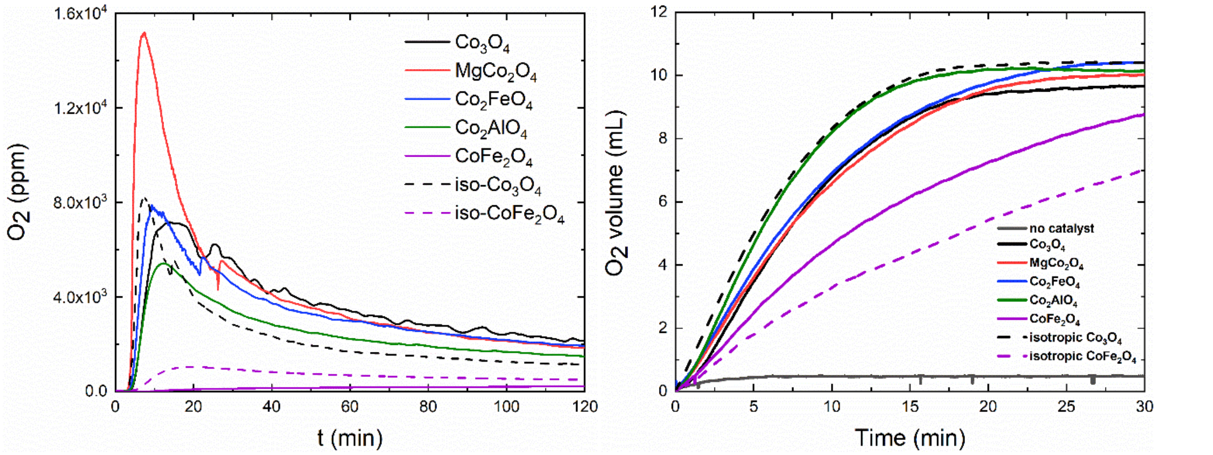

Figure S 18: Oxygen evolution for all catalysts over 120 min for the CAN test (left) and oxygen evolution over 30 min for H_2O_2 decomposition (right).

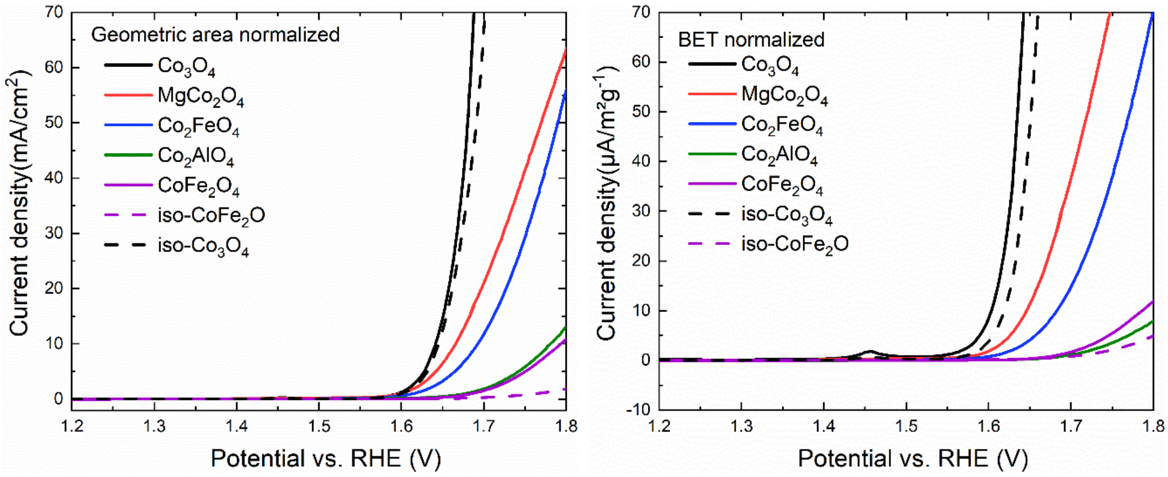

Figure S 19: Current densities as a function of applied potential normalized in the geometric area of the electrode (left) and the BET surface area (right).

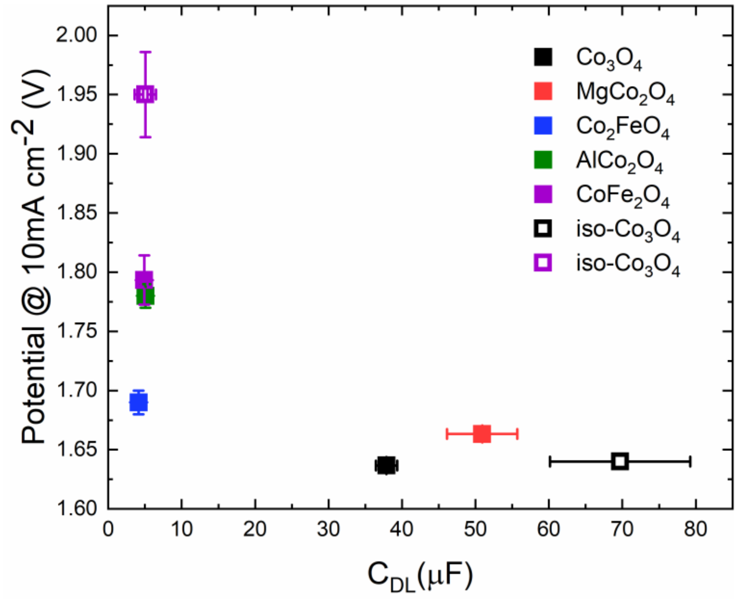

Figure S 20: Double layer capacitance of all samples as a function of the potential at 10 mA cm^{-2}.

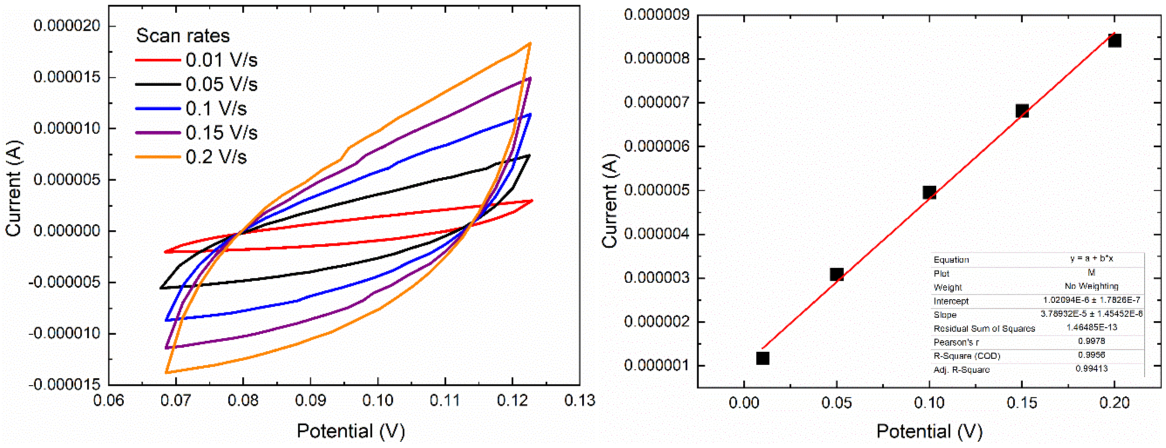

Figure S 21: Cyclic voltammograms with different scan rates (left) and linear regression of the cathodic and anodic potentials.

[1] a) Coronado, J. R. Galan-Mascaros, C. Marti-Gastaldo, A. Ribera, E. Palacios, M. Castro, R. Burriel, *Inorganic chemistry* **2008**, *47*, 9103-9110; b) Li, L. Xing, X. Lu, N. Li, M. Xu, *Inorganic Chemistry Communications* **2015**, *52*, 46-49.

[2] K. F. Ortega, S. Anke, S. Salamon, F. Ozcan, J. Heese, C. Andronescu, J. Landers, H. Wende, W. Schuhmann, M. Muhler, T. Lunkenbein, M. Behrens, *Chem-Eur J* **2017**, *23*, 12443-12449.

[3] J.-M. Génin, S. J. Mills, A. G. Christy, O. Guérin, A. Herbillon, E. Kuzmann, G. Ona-Nguema, C. Ruby, C. Upadhyay, *Mineralogical Magazine* **2014**, *78*, 447-465.

[4] C. Ruby, M. Abdelmoula, S. Naille, A. Renard, V. Khare, G. Ona-Nguema, G. Morin, J.-M. R. Génin, *Geochimica et Cosmochimica Acta* **2010**, *74*, 953-966.

[5] G. Sawatzky, F. Van Der Woude, A. Morrish, *Journal of Applied Physics* **1968**, *39*, 1204-1205.

[6] C. Cannas, A. Musinu, G. Piccaluga, D. Fiorani, D. Peddis, H. K. Rasmussen, S. Mørup, *The Journal of chemical physics* **2006**, *125*, 164714.

[7] a) Q. Wang, G. D. Du, R. Zeng, B. Niu, Z. X. Chen, Z. P. Guo, S. X. Dou, *Electrochim Acta* **2010**, *55*, 4805-4811; b) W. Lou, D. Deng, J. Y. Lee, J. Feng, L. A. Archer, *Advanced Materials* **2008**, *20*, 258-262.

[8] M. J. McKelvy, R. Sharma, A. V. Chizmeshya, R. Carpenter, K. Streib, *Chem Mater* **2001**, *13*, 921-926.
